# Supplementary material for: Cost-effectiveness analysis of COVID-19 booster doses and oral antivirals: Case studies in the Indo-Pacific
Source: PLoS One. 2024 Sep 30;19(9):e0294091. doi: 10.1371/journal.pone.0294091 (PMC11441647; doi:10.1371/journal.pone.0294091)
Supplement: S1 File — Additional detail on the underlying dynamic transmission model (S1), cost-effectiveness parameter estimates (S2), a breakdown of the results (S3), and results from additional scenario analysis (S4). (PDF) [file pone.0294091.s001.pdf]

Cost-effectiveness analysis of COVID-19 booster doses and oral antivirals: case studies in the Indo-Pacific

Supplementary Material

Gizem Mayis Bilgin[1*], Syarifah Liza Munira[2], Kamalini Lokuge[1], and Kathryn Glass[1]

[1] *National Centre for Epidemiology and Population Health, The Australian National University, Acton, ACT 2601, Australia*
[2] *Faculty of Economics and Business, Universitas Indonesia, Jakarta, Indonesia*

[*] *Corresponding author. E-mail address:* gizem.bilgin@anu.edu.au

Table of Contents

S1. Underlying Dynamic Transmission Model 2
S2. Cost-effectiveness Parameter Estimates 4
  S2.1 Estimates of QALYs gained 4
  S2.2 Estimates of productivity losses averted 5
S3. Breakdown of Results 6
S4. Additional Results 12
  S4.1 Deterministic sensitivity analysis for the cost-effectiveness of booster doses 12
  S4.2 Deterministic sensitivity analysis for the cost-effectiveness of oral antivirals 13
  S4.3 Cost-effectiveness of providing oral antivirals to high-risk adults under varying booster programs 14
  S4.4 Cost-effectiveness of providing oral antivirals to different population groups 15
  S4.5 Cost-effectiveness of providing molnupiravir to high-risk adults 16
  S4.6 The impact of antiviral wastage on their likelihood of being cost-effective 17
  S4.7 The impact of long-COVID on the cost-effectiveness of booster doses 18
Reference List 20

S1. Underlying Dynamic Transmission Model

This section provides an overview of the dynamic transmission model underlying this paper's cost-effectiveness analysis. Further detail on the design and execution of the model can be found in a previously published paper which focused on assessing the potential impact of COVID-19 oral antivirals and booster doses in our study settings [1].

The model used a Susceptible-Infected-Exposed-Recovered (SEIR) structure, subdivided by age, comorbidity, and vaccination status by dose and type (Fig S1). Transmission was modelled deterministically, and severe outcomes projected stochastically. Estimates for all basic transmission parameters were kept constant between settings. Differences in transmission dynamics arose from setting-specific differences in age-structure [2], prevalence of comorbidities [3], contact patterns [4], immunity due to previous transmission [3], and vaccine coverage by type and dose [3] (Table S1.1). We estimated vaccine coverage by age and risk groups by first referring to official government announcements (Indonesia [5, 6], Fiji [7-11], Papua New Guinea [12, 13]), national level WHO situational reports (Timor-Leste [14]), and then to the Oxford COVID-19 Government Response Tracker [15].

We stochastically projected severe outcomes by sampling from the underlying estimates of vaccine effectiveness, infection-derived immunity, and antiviral effectiveness against severe outcomes in each simulation. We included vaccine effectiveness against infection, severe disease, and death as dependent on vaccine type (including variation for heterologous mixing of primary schedules with booster doses) and days since vaccination. Infection-derived immunity also included daily time steps to model vaccine effectiveness by time since recovery. Simulations which included oral antivirals considered the likelihood of individuals testing positive within the treatment window, hence, varied the age and immunity profile of the cohort accessing antivirals in each simulation.

Each run of the cost-effectiveness analysis sampled from 100 underlying runs of this dynamic transmission model with replacement. The dynamic transmission model and cost-effectiveness analysis were not run in unison since the transmission model was much more computationally expensive than the cost-effectiveness model, taking hours per run compared to seconds. The dynamic transmission model was particularly computationally expensive due to the number of model compartments included and the daily time-stepped waning of vaccine- and infection-derived immunity.

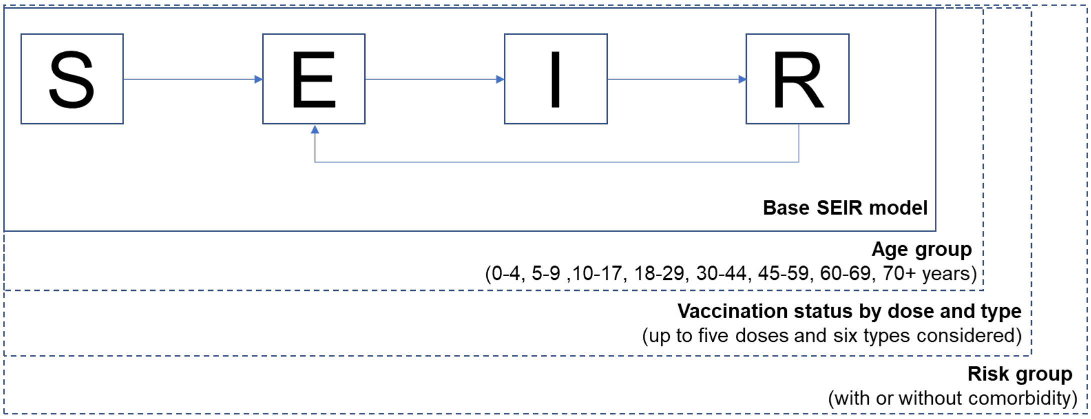

Fig S1. Schematic representing classes of the dynamic transmission model.

Table S1.1 Comparison of key characteristics of study settings.

	World Bank classification [16]	GDP per capita (USD 2022)[16]	Population [2]	Population over 60 (%) [2]	Population at high risk of severe COVID-19 (%) [17]	Primary schedule coverage (%) at 01/01/2023 [3]	Primary schedule coverage in adults aged over 60 (%) at 01/01/2023 [3]
Fiji	Upper middle income	5,317	929,769	9.7	7.1	68.9	96.8
Indonesia	Upper middle income	4,788	275,501,336	10.9	4.7	62.6	50.4
Papua New Guinea	Lower middle income	3,020	10,142,625	5.5	4.7	3.1	2.5
Timor-Leste	Lower middle income	2,358	1,341,298	7.3	3.3	58.9	71.9

S2. Cost-effectiveness Parameter Estimates

S2.1 Estimates of QALYs gained

Table S2.1 Age- and setting-specific estimates of quality-adjusted life years (QALYs) per mild, severe, critical, and fatal COVID-19 case. Estimates for mild, severe, and critical COVID-19 adapted from [18], and estimates for fatal COVID-19 made using life expectancy at each age adjusted by health-related quality of life [2].

Age group	Fiji	Indonesia	Papua New Guinea	Timor-Leste
**QALYs per mild COVID-19 case**				
0 to 4	0.011	0.011	0.011	0.011
5 to 9	0.010	0.010	0.010	0.010
10 to 17	0.010	0.010	0.010	0.010
18 to 29	0.010	0.010	0.010	0.010
30 to 44	0.009	0.009	0.009	0.009
45 to 59	0.009	0.009	0.009	0.009
60 to 69	0.008	0.008	0.008	0.008
70+	0.008	0.008	0.008	0.008
**QALYs per severe COVID-19 case**				
0 to 4	0.021	0.021	0.021	0.021
5 to 9	0.021	0.021	0.021	0.021
10 to 17	0.020	0.020	0.020	0.020
18 to 29	0.020	0.020	0.020	0.020
30 to 44	0.019	0.019	0.019	0.019
45 to 59	0.018	0.018	0.018	0.018
60 to 69	0.017	0.017	0.017	0.017
70+	0.017	0.017	0.017	0.017
**QALYs per critical COVID-19 case (3% discounting of ongoing health complications)**				
0 to 4	4.884	4.884	4.886	4.886
5 to 9	4.642	4.640	4.642	4.642
10 to 17	4.328	4.328	4.328	4.323
18 to 29	3.841	3.840	3.848	3.859
30 to 44	3.191	3.183	3.207	3.224
45 to 59	2.467	2.474	2.488	2.478
60 to 69	1.869	1.869	1.877	1.854
70+	1.293	1.269	1.310	1.289
**QALYs per fatal COVID-19 case (3% discounting of subsequent years of life lost)**				
0 to 4	26.266	26.257	26.119	26.434
5 to 9	25.442	25.427	25.336	25.678
10 to 17	24.174	24.168	24.072	24.450
18 to 29	21.846	21.882	21.840	22.375
30 to 44	17.972	18.033	18.182	18.872
45 to 59	12.787	12.964	13.171	13.731
60 to 69	8.415	8.366	8.598	8.956
70+	4.920	4.560	4.957	5.091

S2.2 Estimates of productivity losses averted

Table S2.2 Age- and setting-specific estimates of productivity losses per severe, critical, and fatal COVID-19 case. Costs are presented in 2022 United States Dollars. The values presented include 3% discounting (baseline assumption) of subsequent years of expected earnings.

Age group	Fiji	Indonesia	Papua New Guinea	Timor-Leste
**Productivity loss per mild COVID-19 case**				
0 to 4	$597.94	$538.06	$488.43	$203.59
5 to 9	$693.88	$625.56	$569.48	$237.30
10 to 17	$828.72	$746.33	$679.75	$284.94
18 to 29	$934.75	$843.81	$764.05	$329.93
30 to 44	$1,622.30	$1,518.46	$1,360.19	$641.50
45 to 59	$1,196.97	$1,237.22	$1,101.91	$586.57
60 to 69	$655.16	$802.88	$651.52	$442.30
70+	$364.28	$449.13	$323.10	$278.05
**Productivity loss per severe COVID-19 case**				
0 to 4	$3,180.90	$2,797.59	$2,620.61	$1,085.23
5 to 9	$3,691.28	$3,252.54	$3,055.45	$1,264.91
10 to 17	$4,340.99	$3,819.20	$3,585.92	$1,498.55
18 to 29	$4,719.72	$4,169.47	$3,890.63	$1,683.92
30 to 44	$11,726.03	$10,849.40	$10,218.14	$4,990.74
45 to 59	$11,355.20	$11,733.33	$10,943.05	$6,106.00
60 to 69	$5,604.62	$6,771.81	$5,720.98	$4,091.50
70+	$3,003.47	$3,597.20	$2,752.11	$2,442.02
**Productivity loss per critical COVID-19 case**				
0 to 4	$20,582.26	$18,102.03	$16,956.88	$7,022.10
5 to 9	$23,884.72	$21,045.88	$19,770.59	$8,184.68
10 to 17	$27,995.07	$24,627.59	$23,118.26	$9,668.31
18 to 29	$30,189.04	$26,677.17	$24,885.45	$10,792.44
30 to 44	$74,467.96	$69,013.48	$64,991.41	$31,803.05
45 to 59	$71,316.85	$74,019.57	$68,765.46	$38,500.98
60 to 69	$34,878.94	$42,241.81	$35,432.24	$25,478.31
70+	$18,489.79	$22,109.69	$16,880.35	$14,945.48
**Productivity loss per fatal COVID-19 case**				
0 to 4	$59,708.01	$54,288.73	$46,606.12	$17,687.11
5 to 9	$80,261.89	$73,160.34	$62,811.24	$23,828.56
10 to 17	$108,588.16	$98,428.50	$84,299.68	$32,610.64
18 to 29	$130,667.42	$118,403.42	$100,398.00	$40,680.05
30 to 44	$120,707.14	$113,304.54	$95,762.05	$42,246.43
45 to 59	$78,037.43	$83,251.70	$68,442.94	$34,420.28
60 to 69	$38,245.81	$48,087.11	$35,359.82	$23,449.10
70+	$20,621.10	$25,434.80	$17,202.83	$14,084.74

S3. Breakdown of Results

Table S3.1 Overview of the net outcomes and costs for different booster dose and oral antiviral eligibility programs. Values presented are expected means from one thousand Monte Carlo simulations. All costs are in 2022 United States Dollars (USD). Default assumptions of 3% discounting for ongoing health outcomes and costs and the use of nirmatrelvir-ritonavir procured at the middle-income reference price ($250 USD per schedule) are used.

Scenario	Health outcomes		Costs		
	Deaths	QALYs lost	Intervention costs	Healthcare costs	Productivity losses
**Fiji**					
Baseline with no booster or oral antiviral program	511	20,698	0	151,513,158	1,746,422,775
No booster, oral antiviral to high-risk adults	259	18,436	19,466,535	123,545,518	1,716,127,041
Booster to high-risk adults, no oral antiviral	225	17,859	438,221	122,601,958	1,698,573,195
Booster to all adults, no oral antiviral	358	18,599	2,117,871	123,496,023	1,671,288,439
Booster to high-risk adults, oral antiviral to high-risk adults	106	16,698	18,095,561	106,281,810	1,681,682,393
Booster to all adults, oral antiviral to high-risk adults	171	16,877	18,008,057	101,330,848	1,647,589,089
**Indonesia**					
Baseline with no booster or oral antiviral program	246,030	6,660,698	0	41,040,795,694	494,555,199,708
No booster, oral antiviral to high-risk adults	118,079	5,614,561	7,818,794,789	31,337,679,588	478,556,693,252
Booster to high-risk adults, no oral antiviral	205,661	6,272,102	74,438,509	37,849,960,623	486,108,085,922
Booster to all adults, no oral antiviral	216,113	6,173,097	476,552,250	36,355,114,362	469,045,374,258
Booster to high-risk adults, oral antiviral to high-risk adults	94,523	5,355,504	7,689,156,251	29,267,420,651	472,009,250,225
Booster to all adults, oral antiviral to high-risk adults	100,250	5,220,002	7,392,973,049	27,468,389,921	454,414,143,498

Scenario	Health outcomes		Costs		
	Deaths	QALYs lost	Intervention costs	Healthcare costs	Productivity losses
**Papua New Guinea**					
Baseline with no booster or oral antiviral program	6,013	171,918	0	1,126,123,047	9,616,702,268
No booster, oral antiviral to high-risk adults	3,216	145,915	113,064,520	962,675,051	9,276,738,567
Booster to high-risk adults, no oral antiviral	6,001	171,799	127,224	1,125,133,724	9,613,428,143
Booster to all adults, no oral antiviral	5,986	171,181	1,427,587	1,114,182,551	9,585,390,432
Booster to high-risk adults, oral antiviral to high-risk adults	3,209	145,838	112,818,218	962,094,863	9,274,129,878
Booster to all adults, oral antiviral to high-risk adults	3,195	145,230	112,726,400	951,197,121	9,246,202,384
**Timor-Leste**					
Baseline with no booster or oral antiviral program	178	6,286	0	32,110,405	194,101,258
No booster, oral antiviral to high-risk adults	100	5,555	6,014,012	28,214,694	187,215,550
Booster to high-risk adults, no oral antiviral	120	5,629	344,689	28,971,686	185,341,050
Booster to all adults, no oral antiviral	121	4,866	2,357,326	24,014,996	154,970,143
Booster to high-risk adults, oral antiviral to high-risk adults	68	5,127	5,424,900	26,191,866	180,488,717
Booster to all adults, oral antiviral to high-risk adults	67	4,352	4,625,881	21,227,327	150,066,662

Table S3.2 Incremental health benefits, costs, and resulting incremental cost-effectiveness ratios (ICERs) for different booster dose and oral antiviral eligibility programs. Values presented are expected means from one thousand Monte Carlo simulations. All costs are in 2022 United States Dollars (USD). Default assumptions of 3% discounting for ongoing health outcomes and costs and the use of nirmatrelvir-ritonavir procured at the middle-income reference price ($250 USD per schedule) are used.

Scenario	Incremental health benefits		Incremental costs			Total incremental cost		ICER	
	Deaths prevents	QALYs gained	Intervention costs	Healthcare costs averted	Productivity losses prevented	Healthcare perspective	Societal perspective	Healthcare perspective	Societal perspective
**Fiji**									
Baseline with no booster or oral antiviral program	0	0	0	0	0	0	0	NA	NA
No booster, oral antiviral to high-risk adults	252	2,262	19,466,535	27,967,640	30,295,734	-8,501,106	-38,796,840	-3,759	-17,155
Booster to high-risk adults, no oral antiviral	286	2,838	438,221	28,911,200	47,849,580	-28,472,979	-76,322,559	-10,031	-26,889
Booster to all adults, no oral antiviral	153	2,099	2,117,871	28,017,136	75,134,336	-25,899,265	-101,033,601	-12,341	-48,142
Booster to high-risk adults, oral antiviral to high-risk adults	405	3,999	18,095,561	45,231,348	64,740,382	-27,135,788	-91,876,170	-6,785	-22,972
Booster to all adults, oral antiviral to high-risk adults	340	3,821	18,008,057	50,182,310	98,833,686	-32,174,253	-131,007,939	-8,421	-34,289
**Indonesia**									
Baseline with no booster or oral antiviral program	0	0	0	0	0	0	0	NA	NA
No booster, oral antiviral to high-risk adults	127,951	1,046,137	7,818,794,789	9,703,116,107	15,998,506,456	-1,884,321,318	-17,882,827,773	-1,801	-17,094
Booster to high-risk adults, no oral antiviral	40,369	388,595	74,438,509	3,190,835,071	8,447,113,786	-3,116,396,562	-11,563,510,348	-8,020	-29,757
Booster to all adults, no oral antiviral	29,917	487,601	476,552,250	4,685,681,332	25,509,825,450	-4,209,129,082	-29,718,954,532	-8,632	-60,949
Booster to high-risk adults, oral antiviral to high-risk adults	151,507	1,305,194	7,689,156,251	11,773,375,043	22,545,949,482	-4,084,218,792	-26,630,168,274	-3,129	-20,403
Booster to all adults, oral antiviral to high-risk adults	145,780	1,440,695	7,392,973,049	13,572,405,773	40,141,056,210	-6,179,432,724	-46,320,488,933	-4,289	-32,151

Scenario	Incremental health benefits		Incremental costs			Total incremental cost		ICER	
	Deaths prevents	QALYs gained	Intervention costs	Healthcare costs averted	Productivity losses prevented	Healthcare perspective	Societal perspective	Healthcare perspective	Societal perspective
**Papua New Guinea**									
Baseline with no booster or oral antiviral program	0	0	0	0	0	0	0	NA	NA
No booster, oral antiviral to high-risk adults	2,797	26,003	113,064,520	163,447,996	339,963,701	-50,383,475	-390,347,177	-1,938	-15,011
Booster to high-risk adults, no oral antiviral	12	119	127,224	989,323	3,274,124	-862,099	-4,136,223	-7,249	-34,781
Booster to all adults, no oral antiviral	27	737	1,427,587	11,940,496	31,311,836	-10,512,909	-41,824,745	-14,266	-56,755
Booster to high-risk adults, oral antiviral to high-risk adults	2,804	26,081	112,818,218	164,028,184	342,572,390	-51,209,966	-393,782,356	-1,964	-15,099
Booster to all adults, oral antiviral to high-risk adults	2,818	26,688	112,726,400	174,925,925	370,499,883	-62,199,525	-432,699,408	-2,331	-16,213
**Timor-Leste**									
Baseline with no booster or oral antiviral program	0	0	0	0	0	0	0	NA	NA
No booster, oral antiviral to high-risk adults	78	731	6,014,012	3,895,711	6,885,708	2,118,301	-4,767,407	2,899	-6,524
Booster to high-risk adults, no oral antiviral	58	658	344,689	3,138,719	8,760,208	-2,794,029	-11,554,237	-4,249	-17,569
Booster to all adults, no oral antiviral	57	1,420	2,357,326	8,095,409	39,131,115	-5,738,083	-44,869,197	-4,040	-31,595
Booster to high-risk adults, oral antiviral to high-risk adults	110	1,160	5,424,900	5,918,538	13,612,541	-493,638	-14,106,180	-426	-12,164
Booster to all adults, oral antiviral to high-risk adults	111	1,934	4,625,881	10,883,078	44,034,596	-6,257,197	-50,291,793	-3,235	-25,999

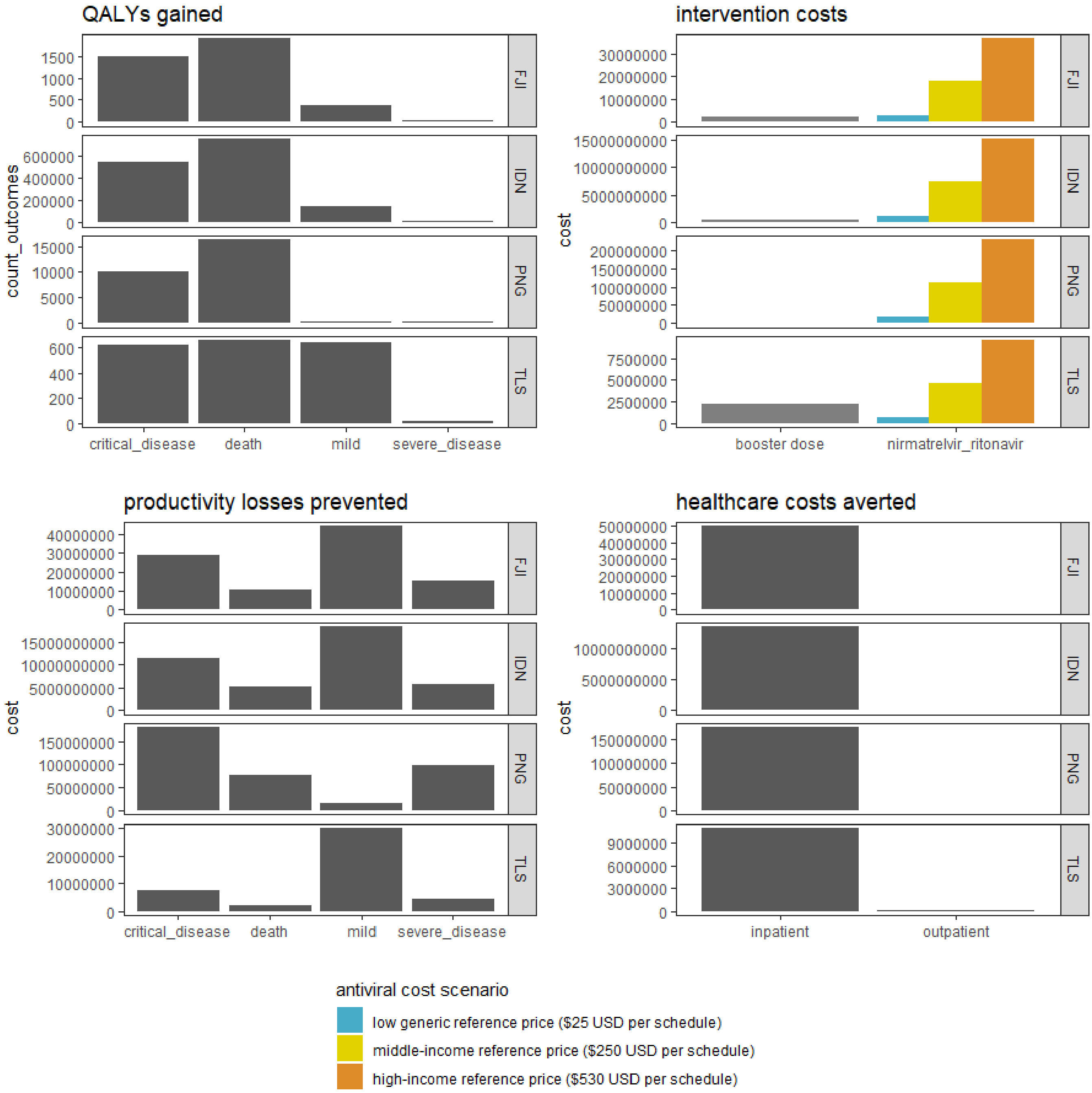

Fig S3.1 Source of quality-adjusted life years (QALYs) gained, intervention costs, productivity losses prevented, and healthcare costs averted for a booster program provided to all adults and oral antiviral to high-risk adults. All costs are in 2022 United States Dollars (USD).

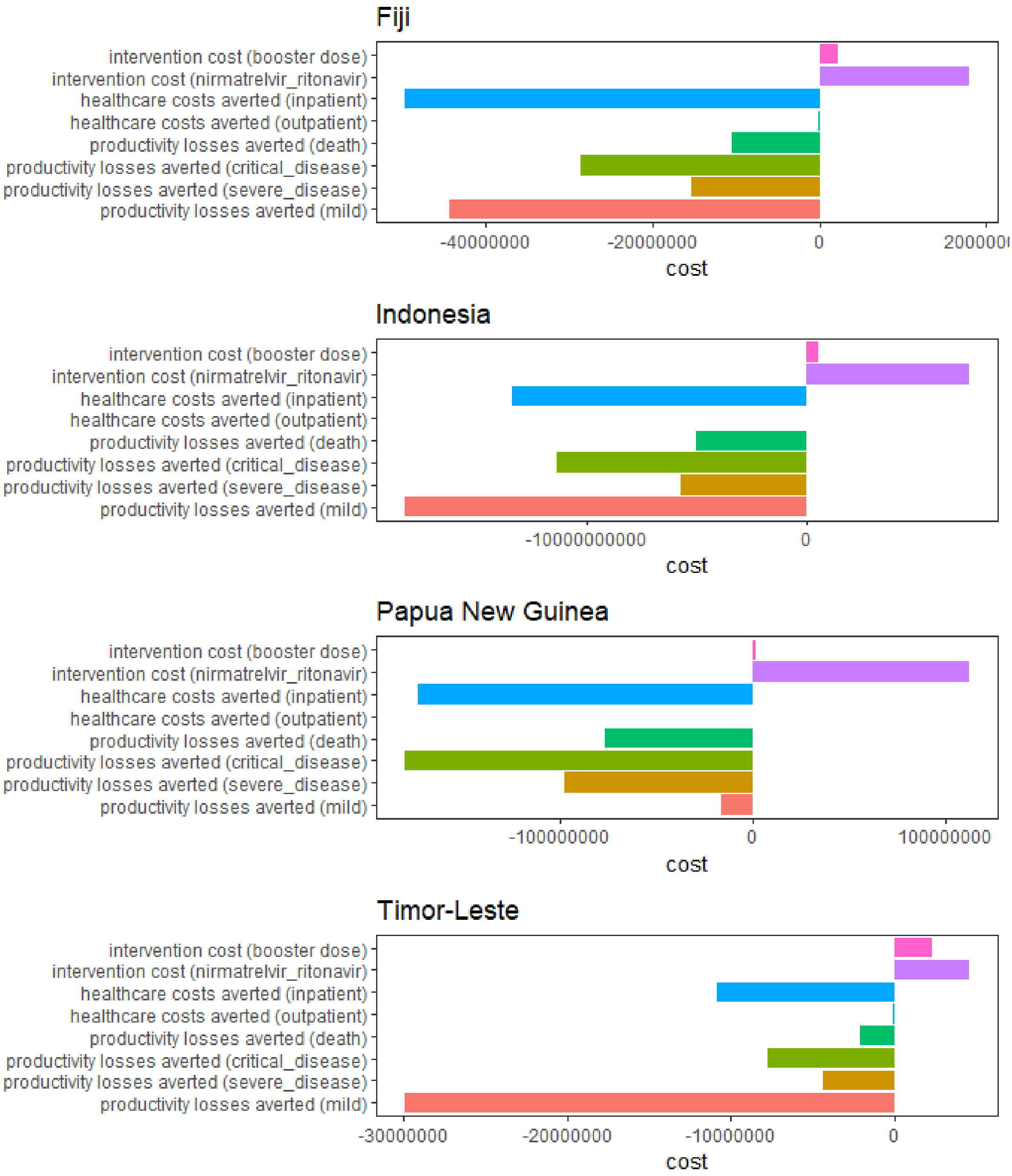

Fig S3.2 Comparison of the size of intervention costs, productivity losses prevented, and healthcare costs averted for a booster program provided to all adults and oral antiviral to high-risk adults. All costs are in 2022 United States Dollars (USD). This figure presents results assuming that oral antivirals are procured at the middle-income reference price ($250 USD per schedule).

S4. Additional Results

An interactive R shiny version of these results can be found hosted on https://gizemmayisbilgin.shinyapps.io/indoPacific_COVID19_costEffectivenessAnalysis/, or directly downloaded from https://github.com/gizembilgin/indoPacific_COVID19_cost_effectiveness/tree/main/03_cost_effectiveness_analysis/07_shiny.

S4.1 Deterministic sensitivity analysis for the cost-effectiveness of booster doses

Figs S4.1 and S4.2 visualise one-way deterministic sensitivity analysis for the influence of the lower and upper estimates for parameters on the cost-effectiveness of booster doses.

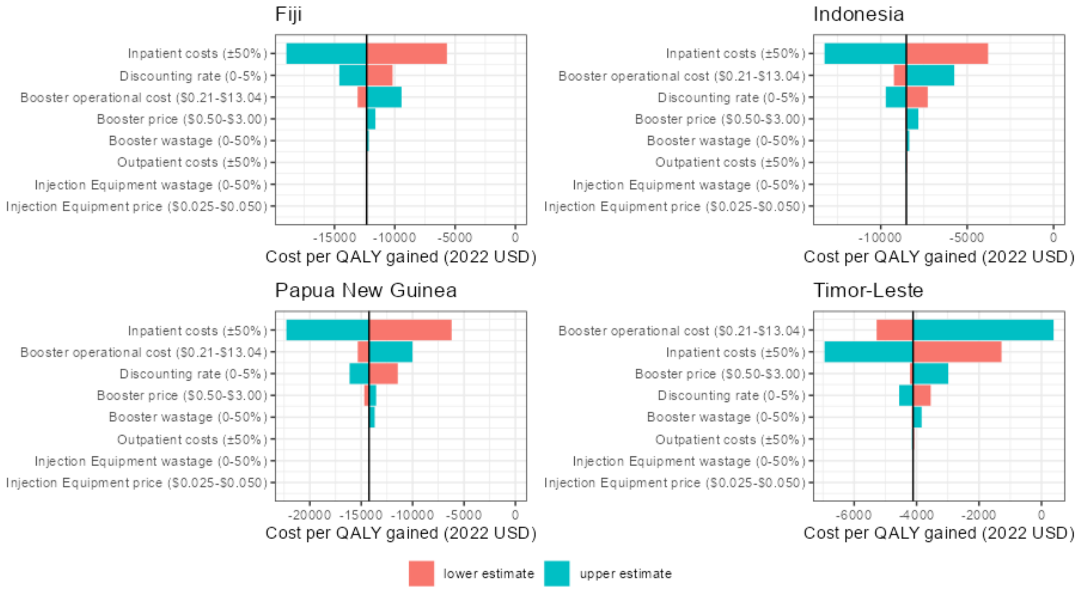

Fig S4.1 Tornado plot visualising deterministic sensitivity analysis of the cost-effectiveness of providing booster doses to all adults in 2023 without oral antivirals from a healthcare perspective

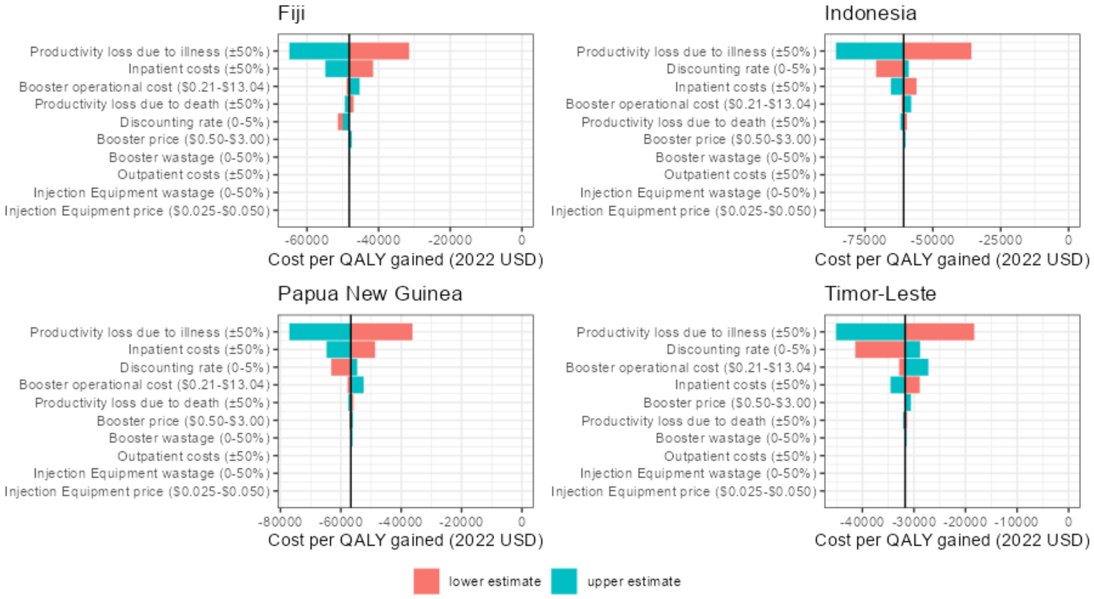

Fig S4.2 Tornado plot visualising deterministic sensitivity analysis of the cost-effectiveness of providing booster doses to all adults in 2023 without oral antivirals from a societal perspective

S4.2 Deterministic sensitivity analysis for the cost-effectiveness of oral antivirals

Figs S4.3 and S4.4 visualise one-way deterministic sensitivity analysis for the influence of the lower and upper estimates for parameters on the cost-effectiveness of oral antivirals.

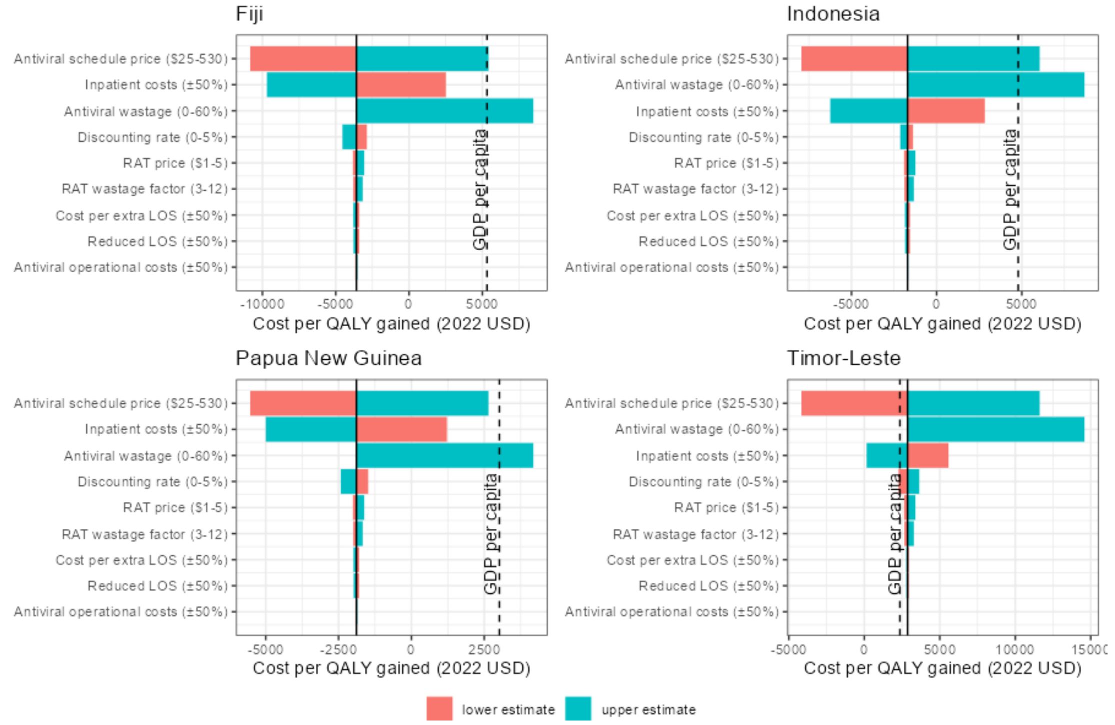

Fig S4.3 Tornado plot visualising deterministic sensitivity analysis of the cost-effectiveness of providing booster doses to nirmatrelvir-ritonavir to high-risk adults in 2023 without further booster doses from a healthcare perspective. The default scenario assumes that oral antivirals are procured at the middle-income reference price and uses 3% discounting.

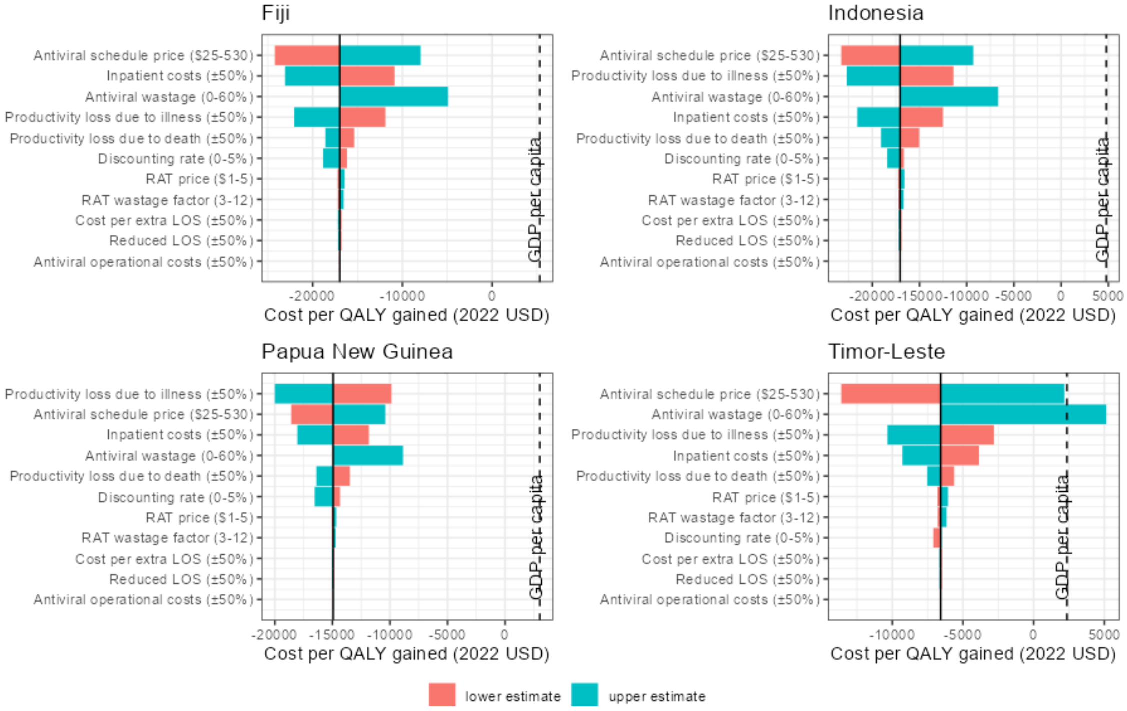

Fig S4.4 Tornado plot visualising deterministic sensitivity analysis of the cost-effectiveness of providing booster doses to nirmatrelvir-ritonavir to high-risk adults in 2023 without further booster doses from a societal perspective. The default scenario assumes that oral antivirals are procured at the middle-income reference price and uses 3% discounting.

S4.3 Cost-effectiveness of providing oral antivirals to high-risk adults under varying booster programs

Fig S4.3 presents a variation on Fig 2 from the main paper, taking a societal perspective instead of a healthcare perspective.

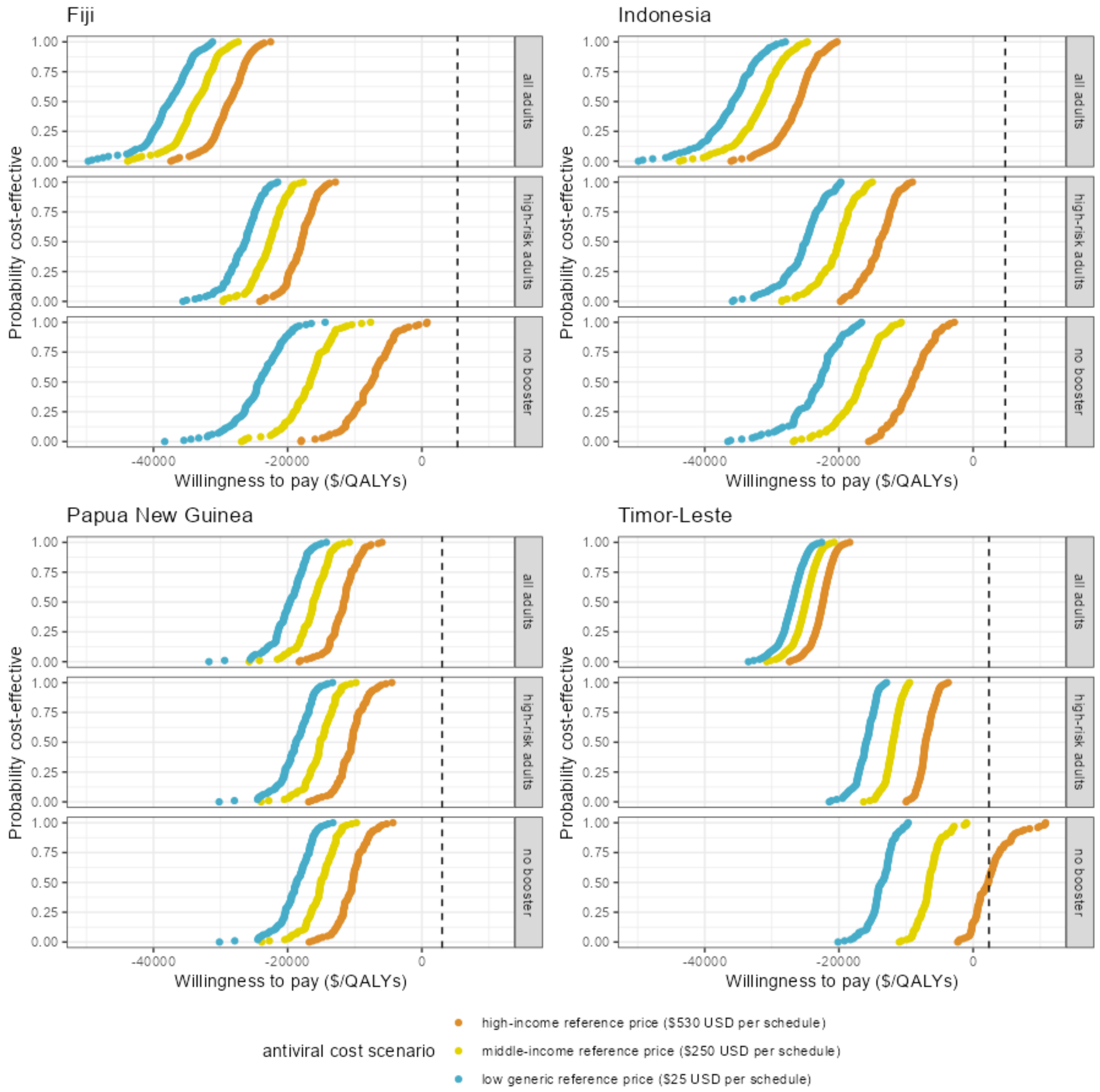

Fig S4.5 Comparison of the probability of an oral antiviral being cost-effective by willingness to pay USD per QALY thresholds across three antiviral schedule prices and three booster eligibility strategies from a societal perspective. One thousand Monte Carlo simulations are presented per scenario. All scenarios assume that oral antivirals are provided to symptomatic high-risk adults. The dashed line represents the nation's gross domestic product per capita.

S4.4 Cost-effectiveness of providing oral antivirals to different population groups

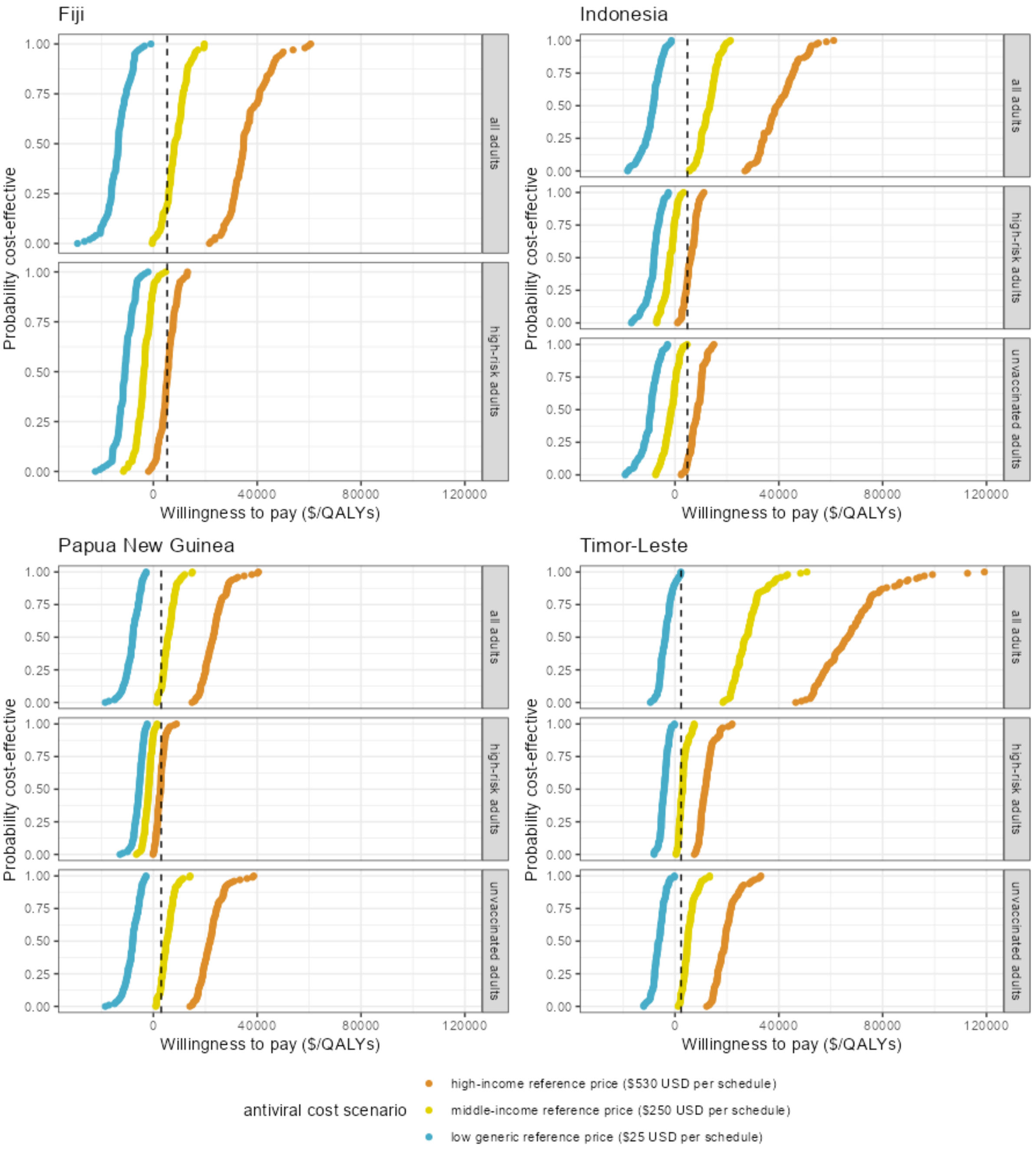

Fig S4.6 Comparison of the probability of an oral antiviral being cost-effective by willingness to pay USD per QALY thresholds across three antiviral schedule prices and three antiviral eligibility strategies from a healthcare perspective. One thousand Monte Carlo simulations are presented per scenario. All scenarios assume no additional booster programs in 2023. The dashed line represents the nation's gross domestic product per capita. We did not conduct simulations with unvaccinated individuals in Fiji since administrative data reports first dose coverage close to 100% in adults [3].

S4.5 Cost-effectiveness of providing molnupiravir to high-risk adults

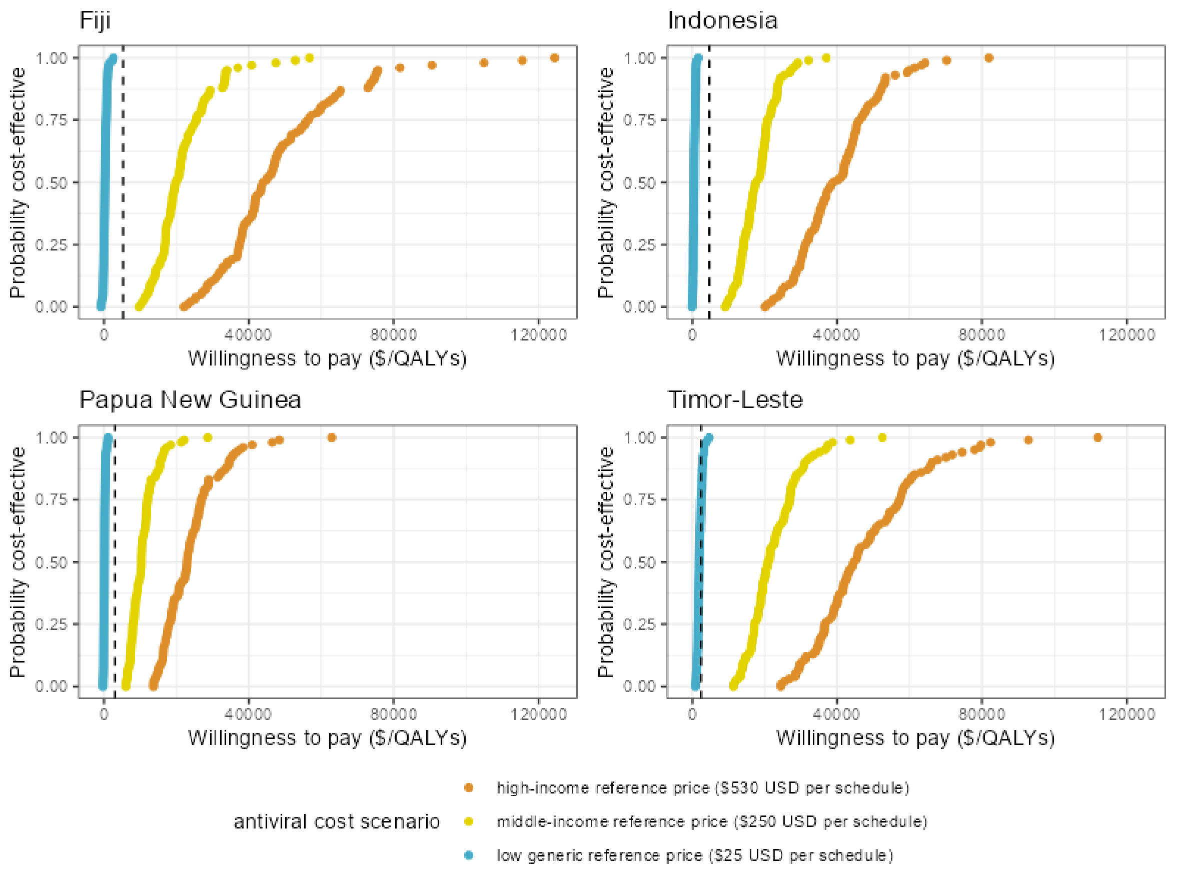

Fig S4.7 Comparison of the probability of providing molnupiravir to symptomatic high-risk adults being cost-effective by willingness to pay USD per QALY thresholds across three antiviral schedule prices from a healthcare perspective. One thousand Monte Carlo simulations are presented per scenario. All scenarios assume no additional booster programs in 2023. The dashed line represents the nation's gross domestic product per capita.

S4.6 The impact of antiviral wastage on their likelihood of being cost-effective

Here we consider the impact of antiviral wastage rates (0%, 20%, 40%, 60%) on the likelihood of providing oral antivirals to high-risk adults being cost-effective. Previous cost-effectiveness analyses have not considered wastage rates of oral antivirals [19-21]. The range presented in Fig S4.8 was informed by a meta-analysis of the proportion of overprescribed or inappropriately dispensed antimicrobials in middle-income countries [22]. At the time of this study, we could not find estimates for real wastage rates of COVID-19 oral antivirals, likely due to the political sensitivity surrounding this topic and their relative novelty.

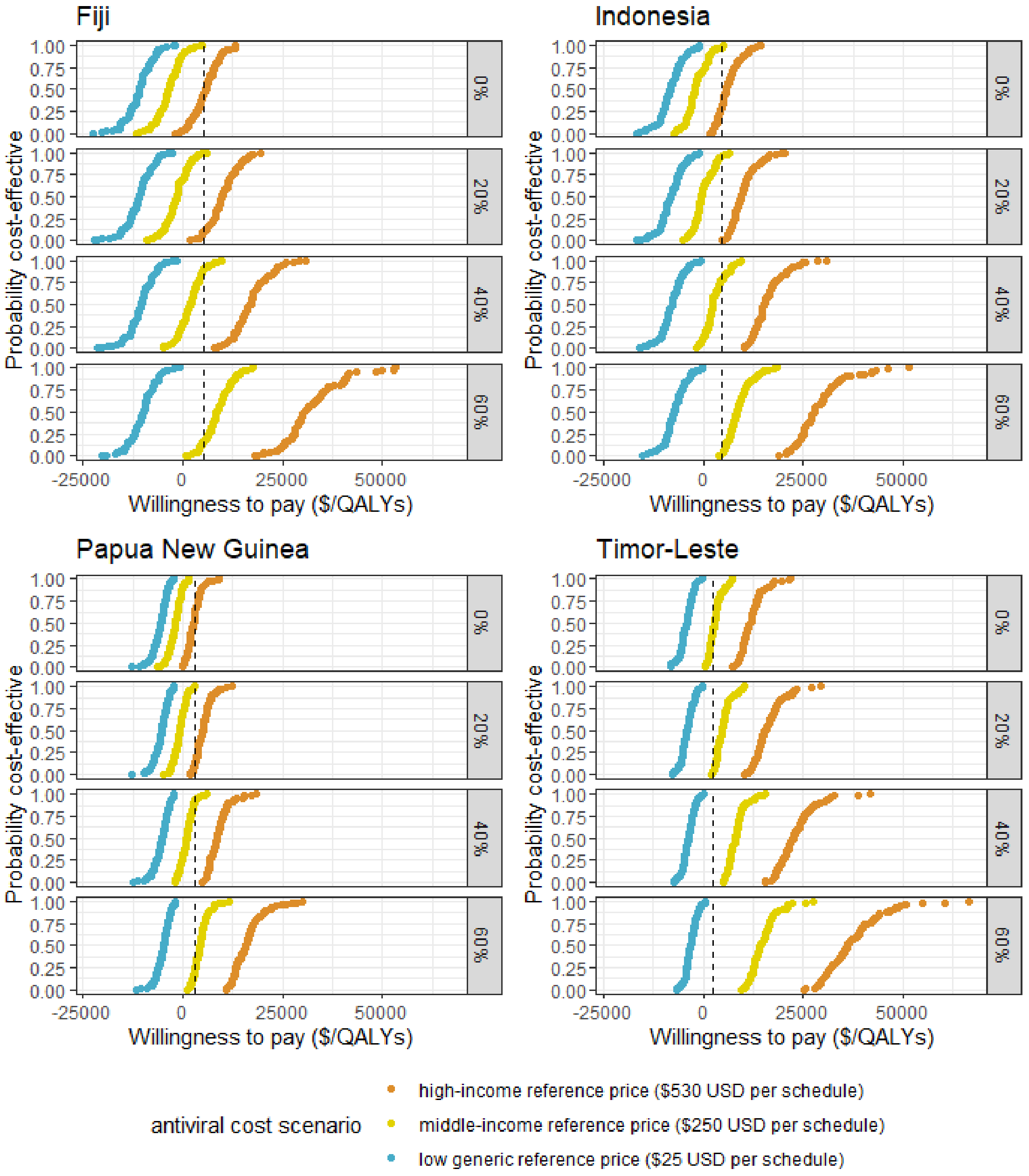

Fig S4.8 The impact of antiviral wastage (0-60%) on the probability of an oral antiviral being cost-effective from a healthcare perspective at different willingness to pay ($/QALY) thresholds. Probabilities have been calculated as a percentage of a thousand Monte Carlo simulations. All scenarios assume that oral antivirals are provided to symptomatic high-risk adults. The dashed line represents the nation's gross domestic product per capita.

S4.7 The impact of long-COVID on the cost-effectiveness of booster doses

Here, we present sensitivity analysis for the inclusion of QALYs associated with long COVID. Two case-control studies have estimated reduced EQ-5D-5L index scores of 0.03 (95% 0.01-0.05) [23] and 0.08 (p = 0.002) [24] for individuals living with long COVID. These estimates roughly align with the disability weight estimate of 0.051 for moderate lower respiratory infections which cause some difficulty with daily activities in the Global Burden of Disease (GBD) Study 2019; hence, we used the GBD estimate for the purpose of this sensitivity analysis [25]. There is great uncertainty in the persistence of long COVID symptoms [26]. Individuals may develop long COVID regardless of whether they experience mild or severe COVID-19 symptoms [27]. We used Indonesian estimates for the prevalence of long COVID – 45.7% between one to six months after infection and 2.7% over six months after infection [28] – notably lower than some estimates from high-income countries such as [29].

Booster doses were cost saving and therefore cost-effective with or without the inclusion of QALYs associated with long COVID (Fig S4.9). Including long COVID increased the QALYs gained by booster programs by 20-85% when high-risk adults were eligible and 140-430% when all adults were eligible (Table S4.1). The inclusion of long COVID had no impact on the cost of booster programs since the we could not include productivity losses associated with long COVID or ongoing healthcare costs associated with long COVID due to an absence of data.

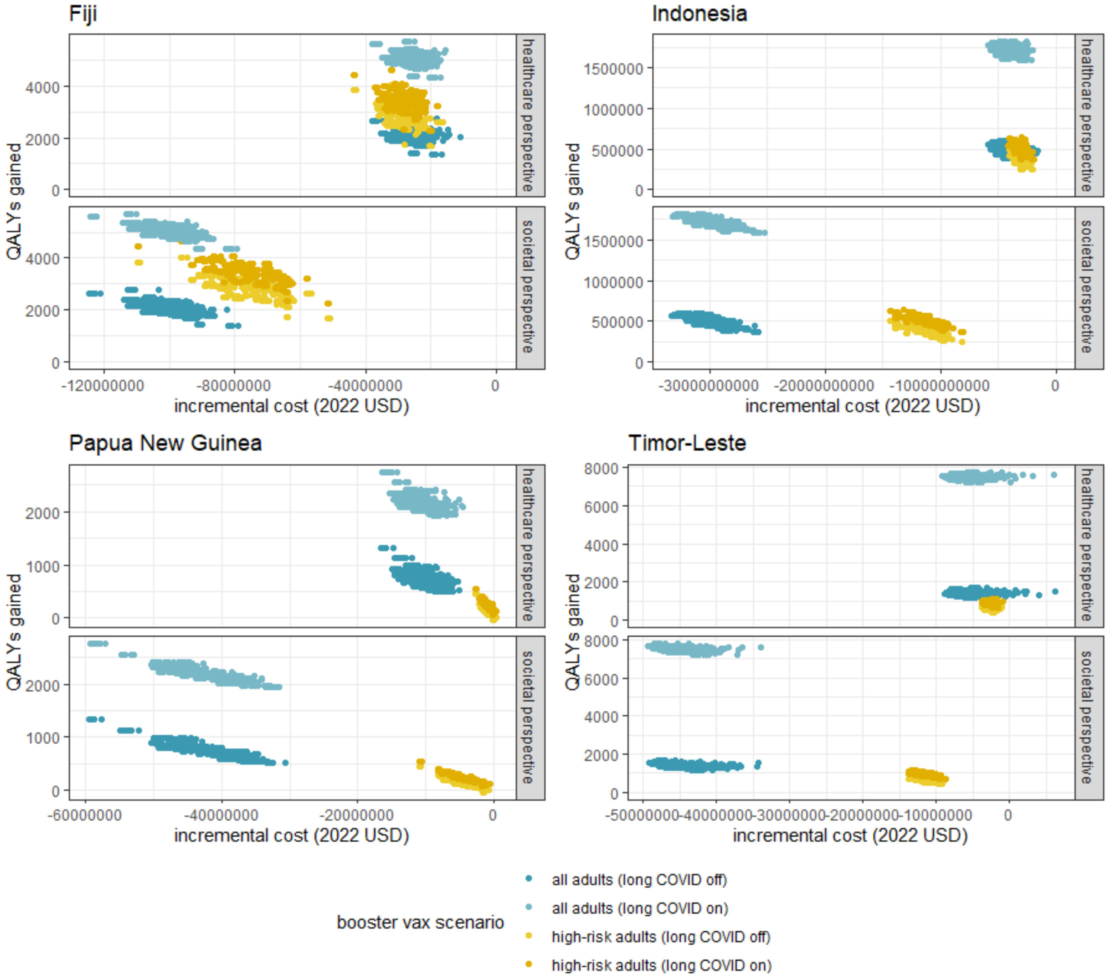

Fig S4.9 The impact of the inclusion of long COVID on the incremental benefits (QALYs) and incremental costs of booster doses in 2023. Each point represents one simulation. One thousand Monte Carlo simulations are represented for each scenario.

Table S4.1 Comparison of the expected mean QALYs gained by setting and booster eligibility scenario with and without long COVID estimates.

Setting	Booster eligibility	QALYs gained (without long COVID)	QALYs gained (including long COVID)
Fiji	all adults	2,090	5,049
Fiji	high-risk adults	2,823	3,429
Indonesia	all adults	487,602	1,709,391
Indonesia	high-risk adults	388,752	515,028
Papua New Guinea	all adults	733	2,161
Papua New Guinea	high-risk adults	119	221
Timor-Leste	all adults	1,417	7,500
Timor-Leste	high-risk adults	655	925

Reference List

1. Bilgin GM, Lokuge K, Munira SL, Glass K. Assessing the potential impact of COVID-19 booster doses and oral antivirals: A mathematical modelling study of selected middle-income countries in the Indo-Pacific. Vaccine: X. 2023;15:100386. doi: 10.1016/j.jvacx.2023.100386.
2. World Population Prospects [Internet]. 2019 [cited 26/11/2021]. Available from: https://population.un.org/wpp/Download/Standard/Mortality/.
3. Dong E, Du H, Gardner L. An interactive web-based dashboard to track COVID-19 in real time. Lancet Infect Dis. 2020;20(5):533-4. doi: 10.1016/S1473-3099(20)30120-1.
4. Prem K, Zandvoort KV, Klepac P, Eggo RM, Davies NG, Centre for the Mathematical Modelling of Infectious Diseases C-WG, et al. Projecting contact matrices in 177 geographical regions: An update and comparison with empirical data for the COVID-19 era. PLoS Comput Biol. 2021;17(7):e1009098. doi: 10.1371/journal.pcbi.1009098.
5. Kementerian Kesehatan Republik Indonesia. Vaksinasi COVID-19 Nasional 2022 [25/01/2023]. Available from: https://vaksin.kemkes.go.id/#/vaccines.
6. Jakarta Smart City. Dashboard Cakupan Vaksinasi Fasilitas Kesehatan di Kecamatan 2023 [updated 22/01/202323/01/2023]. Available from: https://corona.jakarta.go.id/en/cakupan-vaksinasi.
7. Ministry of Health and Medical Services. Nationwide COVID-19 Vaccination Campaign: 28th November 2022 2022 [15/12/2022]. Available from: https://www.health.gov.fj/covid-19-vaccination-campaign/.
8. Ministry of Health and Medical Services. Public Advisory: COVID-19 Vaccine Booster Doses 2022 [15/12/2022]. Available from: https://www.health.gov.fj/booster-doses/.
9. Ministry of Health and Medical Services. COVID-19 Vaccination for Children Aged 15-17 years old: 7th January 2022 2022 [15/12/2022]. Available from: https://www.health.gov.fj/vaccination-schedule-12-17/.
10. Ministry of Health and Medical Services. A second booster dose is available to those eligible 2022 [15/12/2022]. Available from: https://www.health.gov.fj/second-booster-dose/.
11. Ministry of Health and Medical Services. Coronavirus (COVID-19) Vaccines 2022 [15/12/2022]. Available from: https://www.health.gov.fj/covid-vaccine/vaccine-faqs/.
12. National Department of Health. Monday, 31st October 2022 COVID-19 vaccination update Papua New Guinea 2022 [23/12/2022]. Available from: https://covid19.info.gov.pg/Covax%20Updates/COVAX%20update%20%23263_31102022.pdf.
13. National Department of Health. PNG NDOH/NCC COVID-19 Vaccination Dashboard 2022 [23/12/2022]. Available from: https://covid19.info.gov.pg/files/Situation%20Report/NDoH-Vaccination-Dashboard/_COVID-19_VAC_Dashboard-1%20%281%29%2028%20november.pdf.
14. World Health Organization Timor-Leste. Novel Coronavirus (2019-nCoV) situation reports 2022 [18/01/2023]. Available from: https://www.who.int/timorleste/emergencies/novel-coronavirus-2019/novel-coronavirus-(2019-ncov)-situation-reports.
15. Hale T, Angrist N, Goldszmidt R, Kira B, Petherick A, Phillips T, et al. A global panel database of pandemic policies (Oxford COVID-19 Government Response Tracker). Nat Hum Behav. 2021;5(4):529-38. doi: 10.1038/s41562-021-01079-8.
16. The World Bank. World Bank Open Data 2023 [cited 2023 06/07/2023]. Available from: https://data.worldbank.org/.
17. Clark A, Jit M, Warren-Gash C, Guthrie B, Wang HHX, Mercer SW, et al. Global, regional, and national estimates of the population at increased risk of severe COVID-19 due to underlying health conditions in 2020: a modelling study. Lancet Glob Health. 2020;8(8):e1003-e17. doi: 10.1016/S2214-109X(20)30264-3.
18. Robinson LA, Eber MR, Hammitt JK. Valuing COVID-19 Morbidity Risk Reductions. Journal of Benefit-Cost Analysis. 2022;13(2):247-68. doi: 10.1017/bca.2022.11.
19. Insitute for Clinical and Economic Review. Special Assessment of Outpatient Treatments for COVID-19:Final Evidence Report and Meeting Summary. 2022.

20. Jo Y, Kim SB, Radnaabaatar M, Huh K, Yoo JH, Peck KR, et al. Model-based cost-effectiveness analysis of oral antivirals against SARS-CoV-2 in Korea. Epidemiol Health. 2022;44:e2022034. doi: 10.4178/epih.e2022034.
21. Savinkina A, Gonsalves G, Ross JS, Paltiel AD. Determining population-level allocation strategies for COVID-19 treatments in the United States using a quantitative framework, a case study using nirmatrelvir/ritonavir. medRxiv. 2022:2022.08.04.22278431. doi: 10.1101/2022.08.04.22278431.
22. Albarqouni L, Palagama S, Chai J, Sivananthajothy P, Pathirana T, Bakhit M, et al. Overuse of medications in low- and middle-income countries: a scoping review. Bull World Health Organ. 2023;101(1):36-61D. doi: 10.2471/BLT.22.288293.
23. Haberland E, Haberland J, Richter S, Schmid M, Hromek J, Zimmermann H, et al. Seven Months after Mild COVID-19: A Single-Centre Controlled Follow-Up Study in the District of Constance (FSC19-KN). International Journal of Clinical Practice. 2022;2022:8373697. doi: 10.1155/2022/8373697.
24. Tarazona V, Kirouchena D, Clerc P, Pinsard-Laventure F, Bourrion B. Quality of Life in COVID-19 Outpatients: A Long-Term Follow-Up Study. Journal of Clinical Medicine. 2022;11(21). doi: 10.3390/jcm11216478.
25. Vos T, Lim SS, Abbafati C, Abbas KM, Abbasi M, Abbasifard M, et al. Global burden of 369 diseases and injuries in 204 countries and territories, 1990–2019: a systematic analysis for the Global Burden of Disease Study 2019. Lancet. 2020;396(10258):1204-22. doi: 10.1016/S0140-6736(20)30925-9.
26. Huang Q, Jia M, Sun Y, Jiang B, Cui D, Feng L, et al. One-Year Temporal Changes in Long COVID Prevalence and Characteristics: A Systematic Review and Meta-Analysis. Value Health. 2022. doi: 10.1016/j.jval.2022.11.011.
27. Tak CR. The health impact of long COVID: a cross-sectional examination of health-related quality of life, disability, and health status among individuals with self-reported post-acute sequelae of SARS CoV-2 infection at various points of recovery. Journal of Patient-Reported Outcomes. 2023;7(1):31. doi: 10.1186/s41687-023-00572-0.
28. Susanto AD, Isbaniah F, Pratomo IP, Antariksa B, Samoedro E, Taufik M, et al. Clinical characteristics and quality of life of persistent symptoms of COVID-19 syndrome in Indonesia. Germs. 2022;12(2):158-68. doi: 10.18683/germs.2022.1319.
29. Ahmad I, Edin A, Granvik C, Kumm Persson L, Tevell S, Månsson E, et al. High prevalence of persistent symptoms and reduced health-related quality of life 6 months after COVID-19. Frontiers in Public Health. 2023;11. doi: 10.3389/fpubh.2023.1104267.
