# Supplementary material for: Cost-effectiveness analysis of COVID-19 booster doses and oral antivirals: Case studies in the Indo-Pacific
Source: PLoS One. 2024 Sep 30;19(9):e0294091. doi: 10.1371/journal.pone.0294091 (PMC11441647; doi:10.1371/journal.pone.0294091)
Supplement: Attachment — Submitted filename: COVID-19 boosted.docx [file pone.0294091.s002.docx]

I would like to congratulate the authors for this important work. This study gives a good insight into estimation of the cost-effectiveness related to covid-19 vaccine. The authors have done a great work and stated all the analytical assumption behind the outputs. However, there are some major issues that the authors should address:

Major comment

1. The use of QALY derived from VSL which estimated the value of 1 QALY to $580,000. This means the willingness to pay/threshold should be more than $580,000 as per the study by Robinson LA. Estimating the QALY gained by averting mild, moderate, or severe cases of COVID should be done first by a valid HRQOL instrument like EQ5D (https://euroqol.org/support/analysis-tools/index-value-set-calculators/) and then changed to utility score using societal valuation (tariff) for the specific countries. Therefore, the authors need to clarify why they used estimates done by Robinson LA. If this is justified, the study needs alternative analysis method to estimate QALYs.
2. Supplementary file table 2 also mention a huge difference in QALY for mild, severe, fatal cases and critical cases. The QALY values have a huge gap, perhaps due to considering future health states for 2 of the health outcomes and not considering future health states for the other outcomes. Therefore, the authors need to provide the formula on how each of the QALYs for the mild, critical, severe, and fatal condition are calculated.
3. Line 89-95 time horizon of one year is used, if so we do not need to discount using 3%. However, the time horizon looks life time when the supplementary table 2 is seen.

Minor comments

1. The report is well written in detail but it needs restructuring it. To structure the report, please use CHEERS 2022 guideline as it can be a guide for the authors on how to structure this report. this will make it easy for the readers to follow.
2. The paper needs to be corrected for some typos.
3. The result section needs to have numbers reported in it. Example: when doing probabilistic sensitivity analysis, the authors should report the probability of being cost-effective with percentage.
4. In the method section, we need to include the threshold that is used for each country (GDP per capita) and explain why it is preferred.

**Line by line comments**

Abstract: line 3 repeated statement of 3% discount rate.

Abstract: Line 29, 30 A low generic. Do you mean generic price for low-income countries? Please, correct the sentence.

Abstract Line 32 Please, do not use subjective terms like “Interestingly”.

Abstract Line 37: I would recommend not using “continue to be”, as this study will make it look the authors are doing this study for the second time.

Abstract Line 38: “$250 USD”; please correct this to” US$ 250”

Line 59-63: three of the countries are classified as middle-income. Page 4 line 69-73: informs this estimation is for middle-income countries. I recommend the generalizability from this study for middle-income countries, not low-and middle-income countries.

Line 89-95: For a time horizon of one year, we do not need to discount the cost and effect. Discounting will only be applicable for cost and effects that lasts more than one year.

Line 146-47: I suggest all reference estimates to represent middle-income countries only to estimate the operational cost.

Please, write the title of all the figures

Figure 1: please correct figure 1 vertical axis to “incremental QALY gained”, not “QALY averted”

Figure 2: I would suggest not using negative willingness to pay threshold.

Again correct the QALY averted to QALY gained.

Supplementary table 2: in Fiji for age group 0 to 4; QALY= 0.01 for mild, 0.021 for severe, 4.884 for critical cases, and 26.6 for fatal cases.

The above figure looks the QALYs gained for mild, severe, critical, and fatal cases were calculated in different ways. I can see that the QALY for mild and severe are calculated without considering health gains. However, the QALY for critical and fatal cases are calculated using 3% discount rate including the future health gains. Therefore, I strongly recommend for the authors to do the analysis again with the support of senior health economist. In addition, please note that QALY of 1 means the person is in a prefect health and QALY of 0 means the person is dead in a year per person. The authors also need to correct the words “QALY loss” to QALY gain.
