# Supplementary material for: Cost-effectiveness analysis of COVID-19 booster doses and oral antivirals: Case studies in the Indo-Pacific
Source: PLoS One. 2024 Sep 30;19(9):e0294091. doi: 10.1371/journal.pone.0294091 (PMC11441647; doi:10.1371/journal.pone.0294091)
Supplement: Attachment — Submitted filename: Response to Reviewers.docx [file pone.0294091.s003.docx]

We thank the reviewers for their detailed comments. We have clarified sections of our paper and included additional detail based on these comments. We provide line number references in this response letter for the marked-up version of the manuscript with revisions displayed inline.

As per your request, we have also checked the manuscript against PLOS ONE's style requirements, and ensured all figures and tables are referenced within the text.

**Reviewer #1**

**Comment 1 [Abstract]: State how or where the data were derived from.**

We have amended the abstract to state where the data is derived from.

*(Excerpt from the Abstract – line 22)*

*We conducted cost-effectiveness analysis from both healthcare and societal perspectives using data collated from publicly available sources.*

**Comment 2 [Abstract]: Authors stated that the findings support the government program to be cost-effective. This statement sounds like the government make the same cost-effective analysis and the authors compared their findings with that of government's. I believe this is due to writing technicalities. Authors should revise the sentence so that it won't sound that as if the government make the same study.**

We have removed this phrase.

*(Excerpt from the Abstract – line 37)*

*Our results support that ~~government funded~~ COVID-19 booster programs are cost-effective in middle-income settings.*

**Comment 3 [Introduction]: Line 47-48. Data about COVID-19 mortality and morbidity are fluctuative since the disease is newly emerged. Please state when the data was recorded.**

We have included the year in this sentence.

*(Excerpt from the Introduction – line 48)*

*Still, older adults and individuals with comorbidities remain at considerable risk of severe outcomes associated with COVID-19 in 2023 [1, 2].*

**Comment 4 [Introduction]: Authors should provide why it is important to perform cost-effective study, this include its correlation with vaccine acceptance level. (for example, authors may incorporate findings from this study: Narra J. 2022. 2(2): e85)**

We have explained the need to conduct cost-effectiveness analysis.

*(Excerpt from the Introduction – line 67)*

*This paper examines whether COVID-19 booster doses and oral antivirals are cost-effective from healthcare and societal perspectives in Fiji, Indonesia, Papua New Guinea, and Timor-Leste. Understanding the relative efficiency of various interventions is crucial when deciding between competing healthcare priorities. This paper explores the cost-effectiveness of various combinations of booster dose and oral antiviral eligibility.*

We have included a reference to the article suggested by the peer reviewer in our discussion.

*(Excerpt from the Discussion – line 468)*

*Further, if COVID-19 programs transition to private funding than analysis will need to be conducted from a healthcare payer perspective and compared to consumer willingness to pay thresholds (as measured in [3, 4]).*

**Comment 5 [Introduction]: Other than being previously studied (by the same authors), justification on choosing Fiji, Indonesia, etc. should be provided in introduction.**

We have justified our choice of study settings.

*(Excerpt from the Introduction – line 73)*

*Highlighting trends across four middle-income nations with varying vaccine coverage, vaccine hesitancy, prevalence of comorbidities, and age structure extends the relevance of our findings to other middle-income settings. This paper seeks to answer (1) Whether annual booster programs are cost-effective in middle-income settings; (2) Whether oral antiviral programs have the potential to be cost-effective in middle-income settings; and (3) What factors are most influential over whether booster dose and oral antiviral programs are cost-effective in middle-income settings?*

**Comment 6 [Methods]: What do authors mean by societal and healthcare perspectives?**

We have explained what we mean by societal and healthcare perspectives.

*(Excerpt from the Methods – line 93)*

*We present our analyses from both a societal and healthcare perspectives. A healthcare perspective includes only costs borne by the health system; a societal perspective includes all costs regardless of who pays or benefits from them such as the cost of lost productivity due to illness and premature death.*

**Comment 7 [Methods]: Please state how the data were collected in the study design.**

We have stated where data informing the cost-effectiveness analysis was collected from.

*(Excerpt from the Methods – line 89)*

*Here, we estimated the cost of administering COVID-19 booster doses, the cost of delivering COVID-19 oral antivirals, and the costs averted by either intervention using publicly available data.*

**Comment 8 [Methods]: Line 140-142. It is not clear how the assumption was made for the future vaccine products of choice.**

We have clarified this sentence.

*(Excerpt from the Methods – line 156 [previously 140])*

*We assumed that Indonesia, Timor-Leste, and Fiji used Moderna, and Papua New Guinea used Johnson & Johnson for future booster programs based on their previous vaccine supply as documented in UNICEF’s Market Dashboard [5].*

**Comment 9 [Results]: Focus the paragraph to present the data -- not repeating the methods.**

We have read through our results to ensure we are not repeating our methods. We have ensured we remain explicit as to which perspective and prioritisation strategy our cost-effectiveness results are relevant to.

**Comment 10 [Discussion]: Line 380-381. Other than being politically sensitive, some of the countries might suffer from their inability to collect proper data (absence of electronic records for instance or low surveillance for drug misuse).**

We have included this suggestion.

*(Excerpt from the Discussion – line 413 [previously 380])*

*This may be due to the political sensitivity surrounding this data, challenges in collecting these data and/or the relative novelty of oral antivirals in these settings.*

**Comment 11 [Discussion]: Authors mentioned about nirmatrelvir-ritonavir, but before this drugs there have been several antivirals used to treat SARS-CoV-2, please discuss them as well, and how the use of those drugs implicate the present study. (author may incorporate this study: Narra J. 2022. 2(3):e92)**

We have made it explicit in our methods that the results presented in the manuscript are for nirmatrelvir-ritonavir and additional results for molnupiravir are available through the Shiny or in the Supplementary Material.

*(Excerpt from the Methods – line 137)*

*We assumed the use of nirmatrelvir-ritonavir and provided additional results for molnupiravir in the Supplementary Material and R Shiny**.*

Our model was designed to study the cost-effectiveness of antivirals delivered to patients before they develop severe disease. The WHO Therapeutics and COVID-19: Living guideline (January 2023) recommends three types of antivirals for non-severe patients: nirmatrelvir-ritonavir, molnupiravir, and remdesivir [6]. Our paper provides results for nirmatrelvir-ritonavir and molnupirvair. We do not consider remdesivir because it requires intravenous delivery, a delivery mechanism too resource-intensive to implement across all four study settings.

**Comment 12 [Conclusion]: Your findings support the continuation of gov-funded COVID-19 vaccination and procurement of oral anti-SARS-CoV-2. Please make clear about the assumption used in the study, and how the findings are not generalizable. For example, data about COVID-19 fatality and transmission is keep changing through times (if not months). Will your study still relevant if the fatality/transmission rate changes?**

We have included an additional sentence in the conclusion to emphasise that the relevance of our results will change over time.

*(Excerpt from the Conclusion – line 490)*

*Future rollout of both interventions should monitor their wastage rates, the cost of care for patients hospitalised with COVID-19, and emerging evidence on the impacts of long COVID. The relative cost-effectiveness of investing in COVID-19 booster doses and oral antivirals compared to the opportunity costs of investing in other healthcare interventions will vary as the levels of transmission and severity of COVID-19 change over time.*

**Reviewer #2:**

**I would like to congratulate the authors for this important work. This study gives a good insight into estimation of the cost-effectiveness related to covid-19 vaccine. The authors have done a great work and stated all the analytical assumption behind the outputs. However, there are some major issues that the authors should address:**

**Comment 1: The use of QALY derived from VSL which estimated the value of 1 QALY to $580,000. This means the willingness to pay/threshold should be more than $580,000 as per the study by Robinson LA. Estimating the QALY gained by averting mild, moderate, or severe cases of COVID should be done first by a valid HRQOL instrument like EQ5D (https://euroqol.org/support/analysis-tools/index-value-set-calculators/) and then changed to utility score using societal valuation (tariff) for the specific countries. Therefore, the authors need to clarify why they used estimates done by Robinson LA. If this is justified, the study needs alternative analysis method to estimate QALYs.**

We have checked that the Robinson, Eber & Hammitt paper used health-related quality of life (HRQoL) estimates to translate mild, moderate, and severe cases of COVID-19 into the QALY values used by our model [7]. We used these estimates as one of the first published papers valuing QALYs associated with nonfatal cases of COVID-19. Research valuing COVID-19 morbidity is still emerging.

We used Robinson et al.’s estimates for QALYs but not their estimates on the monetary values of nonfatal cases in our model for two reasons. Firstly, Robinson’s estimate of $580,000 for the value of a statistical life (VSL) was for an American context and would not be appropriate in our four middle-income settings. Secondly, we conducted cost-effectiveness analysis not cost-benefit analysis, so we did not monetise outcomes in our study. The WHO identifies cost-effectiveness analysis as the preferred method for evaluating vaccine programs [8].

We have included additional detail on our choice of willingness to pay thresholds in response to Reviewer 2 Comment 7.

**Comment 2: Supplementary file table 2 also mention a huge difference in QALY for mild, severe, fatal cases and critical cases. The QALY values have a huge gap, perhaps due to considering future health states for 2 of the health outcomes and not considering future health states for the other outcomes. Therefore, the authors need to provide the formula on how each of the QALYs for the mild, critical, severe, and fatal condition are calculated.**

The peer reviewer is correct, there is a large gap between mild and severe cases and critical and fatal cases because mild and severe cases do not include ongoing effects. This is deliberate because, by their definition, mild and severe cases do not experience ongoing health complications.

We have included the source of our estimates for QALYs per mild, severe, critical, and fatal COVID-19 in the caption of Supplementary Table S2.1:

*(Excerpt from the Supplementary Material – page 4)*

***Table S2.1 Age- and setting-specific estimates of quality-adjusted life years (QALYs) per mild, severe, critical, and fatal COVID-19 case. Estimates for mild, severe, and critical COVID-19 adapted from [7] and estimates for fatal COVID-19 made using life expectancy at each age adjusted by health-related quality of life [9].***

We provided further detail in the manuscript:

*(Excerpt from the Methods – line 141)*

***Translating health outcomes to QALYs***

*We calculated quality-adjusted life years (QALYs) saved by booster doses and/or oral antivirals by considering the number of deaths prevented, and incidence of nonfatal mild, severe, and critical COVID-19 averted. We adapted estimates by Robinson, Eber & Hammitt (2022) to translate the incidence of mild, severe, and critical COVID-19 to QALYs [7] (Table S2.1). This study by Robinson et al. considered the duration of symptoms and health-related quality of life (HRQoL) of nonfatal symptomatic cases. We converted deaths prevented into QALYs using life expectancy at each age from United Nations World Prospect Population estimates for 2022 [9], adjusted by the same age specific HRQoL estimates as in [7]. We considered QALYs associated with long COVID for additional sensitivity analyses presented in S4.7 of the Supplementary Material.*

We have ensured that the script used to import QALY estimates into our model is thoroughly commented, and available online: <https://github.com/gizembilgin/indoPacific_COVID19_cost_effectiveness/blob/main/03_cost_effectiveness_analysis/03_mech_shop/(mech%20shop)%20estimate%20QALYs%20per%20outcome.R>

**Comment 3: Line 89-95 time horizon of one year is used, if so we do not need to discount using 3%. However, the time horizon looks life time when the supplementary table 2 is seen.**

We have clarified that we modelled the effects of booster doses and oral antivirals on COVID-19 incidence within a year but included the ongoing morbidity associated with this incidence. This involved considered the reduced quality of life and productivity losses due to lifelong disability or death associated with critical and fatal COVID-19 cases occurring within the one year time frame.

*(Excerpt from the Methods – line 101)*

*We estimated the impact of booster doses and oral antivirals on the incidence of severe disease over the time horizon of one year. We included ongoing morbidity and productivity losses associated with cases occurring within this year; these ongoing effects occur across the lifetime of the individual and were discounted by 3%. The choice to use a time horizon of one year aligned with existing economic modelling for COVID-19, see systematic review [10]. A one-year time horizon was appropriate for the evaluation of oral antivirals since the effects of oral antivirals are short-term and localised to the individual receiving the antivirals. A one-year time horizon was conservative but appropriate for the evaluation of booster doses since the increased protection against severe outcomes provided by booster doses compared to previous vaccination was expected to wane after one year [11].*

**[Reviewer 2] Minor comments:**

**Comment 4: The report is well written in detail but it needs restructuring it. To structure the report, please use CHEERS 2022 guideline as it can be a guide for the authors on how to structure this report. this will make it easy for the readers to follow.**

We have checked our manuscript against the 28-item CHEERS guidelines (see below). We have renamed headings and reordered parts of our Methods so that they align better with the order in which the items are listed in the CHEERS guidelines.

| CHEERS item number | Manuscript location |
| --- | --- |
| 1 – Title | Title on lines 4-5 |
| 2 – Abstract | Abstract on lines 15-41 |
| 3 – Background and Objectives | Covered in the Introduction with background on lines 44-65 and objectives on lines 67-79 |
| 4 – Health economic analysis plan | Not applicable as this study does not accompany an RCT |
| 5 – Study population  6 – Setting and location  7 – Comparators  8 – Perspective  9 – Time horizon  10 – Discount rate | Covered in Methods under heading ‘study design’ with items 5 & 6 on lines 83-89, item 7 on lines 91-93, item 8 on lines 93-97, item 9 on lines 101-111, and item 10 on line 102-105 |
| 11 – Selection of outcomes  12 – Measurement of outcomes  13 – Valuation of outcomes | Covered in Methods under heading ‘health outcomes’ with item 11 on lines 114-124, item 12 on lines 126-139, and item 13 on lines 141-151 |
| 14 – Measurement and valuation of resources and costs  15 – Currency, price data, and conversion | Covered in Methods under headings ‘intervention costs’ and ‘healthcare and productivity costs averted’ from lines 153 to 245 |
| 16 – Rationale and description of model | Provided on lines 82-124 |
| 17 – Analytics and assumptions  18 – Characterizing heterogeneity  19 – Characterizing distributional effects | Included throughout Methods where relevant to the assumptions made for setting-specific estimation of health outcomes and costs |
| 20 – Characterizing uncertainty | Covered in Methods under heading ‘sensitivity analysis to characterise uncertainty’ on lines 247-258 |
| 21 – Approach to engagement with patients and others affected by the study | Not applicable |
| 22 – Study parameters | Estimates provided throughout Methods in health outcome and cost sections |
| 23 – Summary of main results | Covered at the start of each sub-section in the results: lines 276-289, lines 321-329, lines 368-373 |
| 24 – Effect of uncertainty | Covered at the end of each sub-section in the results: lines 297-312, lines 339-357, lines 373-378 |
| 25 – Effect of engagement with patients and other affected by the study | Not applicable |
| 26 – Study findings, limitations, generalisability, and current knowledge | Our Discussion has been structured in this order: study findings on lines 388-451, limitations on lines 453-484, generalisability and current knowledge in the Conclusion on lines 487-495 |
| 27 – Source of funding | Line 505 |
| 28 – Conflicts of interest | Line 506 |

**Comment 5: The paper needs to be corrected for some typos.**

We have thoroughly read through the manuscript and corrected a few typos.

**Comment 6: The result section needs to have numbers reported in it. Example: when doing probabilistic sensitivity analysis, the authors should report the probability of being cost-effective with percentage.**

We have considered where numbers would support the text in the results and included numbers under the results sub-heading ‘cost-effectiveness of oral antivirals’:

*(Excerpt from Results – line 321)*

*Oral antiviral programs for high-risk adults demonstrated the potential to be cost-effective in all settings using Pfizer’s agreed low generic price, were cost-effective in three settings (not Timor-Leste, only 41% likelihood) using the middle-income reference price, and in one setting (Papua New Guina with 61% likelihood) using the high-income reference price from a healthcare perspective with GDP per capita as a threshold for cost-effectiveness (Fig 2). In comparison, from a societal perspective, providing oral antivirals to high-risk adults was cost saving in three settings (not Timor-Leste), and cost-effective in all settings even when using the high-income reference price (53% likelihood in Timor-Leste and 100% in three other settings) (Figure S4.5).*

**Comment 7: In the method section, we need to include the threshold that is used for each country (GDP per capita) and explain why it is preferred.**

We have included the threshold for cost-effectiveness in the methods and added a few sentences in the discussion on the limitations of this cost-effectiveness threshold.

*(Excerpt from the Methods – line 84)*

*We used GDP per capita as a default cost-effectiveness threshold: Fiji (US$5,317), Indonesia (US$4,788), Papua New Guinea (US$3,020), Timor-Leste (US$2,358) [12].*

*(Excerpt from the Discussion – line 464)*

*We used GDP per capita as a default threshold for cost-effectiveness. The limitations of this approach have been clearly discussed in literature [13-15]. Contextual factors such as competing financial demands and healthcare capacity will determine a decision maker’s willingness to pay per QALY averted by a COVID-19 intervention.*

**[Reviewer 2] Line by line comments:**

**Comment 8: Abstract Line 3 repeated statement of 3% discount rate.**

We have removed this repeated statement.

**Comment 9: Abstract Line 29, 30 A low generic. Do you mean generic price for low-income countries? Please, correct the sentence.**

We have updated the sentence to read “at a low generic price”. Here, we are referring to the lower price anticipated for generic versions of oral antivirals.

**Comment 10: Abstract Line 32 Please, do not use subjective terms like “Interestingly”.**

We have removed this subjective phrase.

**Comment 11: Abstract Line 37: I would recommend not using “continue to be”, as this study will make it look the authors are doing this study for the second time.**

We have improved the wording from “continue to be” to “are”.

**Comment 12: Abstract Line 38: “$250 USD”; please correct this to” US$ 250”**

We have made this change and corrected all references to USD throughout the manuscript.

**Comment 13: Line 59-63: three of the countries are classified as middle-income. Page 4 line 69-73: informs this estimation is for middle-income countries. I recommend the generalizability from this study for middle-income countries, not low-and middle-income countries.**

We agree with the reviewer and have removed the mention of low-income countries in this line. Our results are generalisable to middle-income countries, not low- and middle-income countries, since all four of our settings are middle-income countries.

**Comment 14: Line 89-95: For a time horizon of one year, we do not need to discount the cost and effect. Discounting will only be applicable for cost and effects that lasts more than one year.**

Our description of the model’s time horizon was clarified in our response to Reviewer 2’s third comment.

**Comment 15: Line 146-47: I suggest all reference estimates to represent middle-income countries only to estimate the operational cost.**

We have checked whether a middle-income country estimate is available. Unfortunately, the meta-analysis used to inform this model parameter did not provide an estimate for middle-income countries alone. We conducted sensitivity analysis with a broad range ($0.21 to $13.04) around the estimate for low- and middle-income countries ($2.85). Sensitivity analysis demonstrated that this parameter was not influential over the incremental cost-effectiveness of booster doses (Fig 3).

**Comment 16: Figure 1: please correct figure 1 vertical axis to “incremental QALY gained”, not “QALY averted”.**

We have corrected the wording to “QALYs gained” in this figure and in all other references throughout the manuscript and Supplementary Material.

**Comment 17: Figure 2: I would suggest not using negative willingness to pay threshold.**

The willingness to pay thresholds used in Figure 2 are positive. The x-axis of willingness to pay includes negative values because there is a probability that these interventions are cost-saving.

**Comment 18: Supplementary table 2: in Fiji for age group 0 to 4; QALY= 0.01 for mild, 0.021 for severe, 4.884 for critical cases, and 26.6 for fatal cases. The above figure looks the QALYs gained for mild, severe, critical, and fatal cases were calculated in different ways. I can see that the QALY for mild and severe are calculated without considering health gains. However, the QALY for critical and fatal cases are calculated using 3% discount rate including the future health gains. Therefore, I strongly recommend for the authors to do the analysis again with the support of senior health economist. In addition, please note that QALY of 1 means the person is in a prefect health and QALY of 0 means the person is dead in a year per person. The authors also need to correct the words “QALY loss” to QALY gain.**

We have addressed this comment in our responses to Reviewer 2’s Comment 2 and 16.

In summary, we used estimates for nonfatal morbidity of COVID-19 adapted from ‘Valuing COVID-19 Morbidity Risk Reductions’ by Robinson, Eber & Hammitt [7]. This study estimated morbidity of 0.01 QALYs per mild, 0.02 QALYs per severe, 3.15 QALYs per critical COVID-19 case (estimate without age-disaggregation). The peer reviewer is correct; mild and severe cases do not include ongoing effects, and hence their calculation does not involve discounting. This is deliberate because mild and severe cases do not experience ongoing health complications by their definition.

We have corrected the wording to “QALYs gained” or “QALYs saved” throughout the manuscript and Supplementary Material.

**Reference List**

1.	World Health Organization. WHO SAGE roadmap on uses of COVID-19 vaccines in the context of Omicron and substantial population immunity: an approach to optimize the global impact of COVID-19 vaccines at a time when Omicron and its sub-lineages are the dominant circulating variants of concern, based on public health goals, evolving epidemiology, and increasing population-level immunity, first issued 20 October 2020, updated: 13 November 2020, updated: 16 July 2021, update: 21 January 2022, latest update: 30 March 2023. Geneva: World Health Organization, 2023 Contract No.: WHO/2019-nCoV/Vaccines/SAGE/Prioritization/2023.1.

2.	Sasanami M, Fujimoto M, Kayano T, Hayashi K, Nishiura H. Projecting the COVID-19 immune landscape in Japan in the presence of waning immunity and booster vaccination. Journal of Theoretical Biology. 2023;559:111384. doi: 10.1016/j.jtbi.2022.111384.

3.	Viveiros Rosa S, Rayhan M, Mudatsir M, Nurjannah N, Ichsan I, Amir-Behghadami M, et al. Acceptance and willingness to purchase a hypothetical COVID-19 vaccine in a region under Shariah law: A cross-sectional study in Aceh, Indonesia. 2022;2:7. doi: 10.52225/narra.v2i2.85.

4.	Harapan H, Wagner AL, Yufika A, Winardi W, Anwar S, Gan AK, et al. Willingness-to-pay for a COVID-19 vaccine and its associated determinants in Indonesia. Human Vaccines & Immunotherapeutics. 2020;16(12):3074-80. doi: 10.1080/21645515.2020.1819741.

5.	United Nation's Children's Fund. COVID-19 Market Dashboard 2022 [Cited 15/12/2022]. Available from: https://www.unicef.org/supply/covid-19-market-dashboard.

6.	World Health Organization. Therapeutics and COVID-19: living guideline, 13 January 2023. Geneva: World Health Organization, 2023 Contract No.: WHO/2019-nCoV/therapeutics/2023.1.

7.	Robinson LA, Eber MR, Hammitt JK. Valuing COVID-19 Morbidity Risk Reductions. Journal of Benefit-Cost Analysis. 2022;13(2):247-68. doi: 10.1017/bca.2022.11.

8.	World Health Organization. WHO guide for standardization of economic evaluations of immunization programmes. Geneva: World Health Organization, 2019 Contract No.: WHO/IVB/19.10.

9.	World Population Prospects 2019 [Cited 26/11/2021]. Available from: https://population.un.org/wpp/Download/Standard/Mortality/.

10.	Izadi R, Hatam N, Baberi F, Yousefzadeh S, Jafari A. Economic evaluation of strategies against coronavirus: a systematic review. Health Economics Review. 2023;13(1):18. doi: 10.1186/s13561-023-00430-1.

11.	International Vaccine Access Center, World Health Organization. Results of COVID-19 Vaccine Effectiveness Studies: An Ongoing Systematic Review. 2022.

12.	The World Bank. World Bank Open Data [Cited 06/07/2023]. Available from: https://data.worldbank.org/.

13.	Bertram MY, Lauer JA, De Joncheere K, Edejer T, Hutubessy R, Kieny MP, et al. Cost-effectiveness thresholds: pros and cons. Bull World Health Organ. 2016;94(12):925-30. doi: 10.2471/blt.15.164418.

14.	Marseille E, Larson B, Kazi DS, Kahn JG, Rosen S. Thresholds for the cost-effectiveness of interventions: alternative approaches. Bull World Health Organ. 2015;93(2):118-24. doi: 10.2471/blt.14.138206.

15.	Newall AT, Jit M, Hutubessy R. Are Current Cost-Effectiveness Thresholds for Low- and Middle-Income Countries Useful? Examples from the World of Vaccines. PharmacoEconomics. 2014;32(6):525-31. doi: 10.1007/s40273-014-0162-x.
