# Supplementary material for: Cost-effectiveness analysis of COVID-19 booster doses and oral antivirals: Case studies in the Indo-Pacific
Source: PLoS One. 2024 Sep 30;19(9):e0294091. doi: 10.1371/journal.pone.0294091 (PMC11441647; doi:10.1371/journal.pone.0294091)
Supplement: Attachment — Submitted filename: 2024_07_01 response to reviewers.docx [file pone.0294091.s004.docx]

We thank the reviewers for taking to the time to provide feedback on our revised manuscript. We have further clarified our paper to demonstrate reproducibility and address concerns surrounding the presentation of our methods and results. We provide line number references in this response letter for the marked-up version of the manuscript with revisions displayed inline.

**Reviewer #1**

**Thank you for addressing my previous concerns. However, authors stated that the data were retrieved from 'publicly available sources'. This is not reproducible and convincing. Authors should provide more detail and at least use several criteria for the data. Some countries might not appropriately collect their data, hence doubt on the methodology.**

We provide the data used by the model in the *01_inputs* folders of our GitHub repository as .csv, .Rdata, and .xlsx files. We explicitly reference and provide weblinks to data sources in our paper and supplementary material. Providing the data and code used by the model ensures our results are reproducible; providing weblinks to data sources ensures our results can be updated by future users.

We have amended the data availability statement to better reflect the reproducibility of our results:

*(Excerpt from the manuscript – line 485)*

*All data was collated from publicly available sources.* *References to data sources are provided in the paper and supplementary material. To ensure reproducibility, model code and data inputs are provided on GitHub: <https://github.com/gizembilgin/indoPacific_COVID19_cost_effectiveness/tree/main>.*

**Reviewer #2:**

**Comment 1: Line 95-99: Response not addressed properly from previous comment 3. The authors are not providing a clear response. The authors should either do the analysis with 1 year timeframe without considering future morbidity (consequences in terms both cost and outcome) or multiple year by discounting future consequences (for both the cost and outcome). In economic evaluation, It is methodologically incorrect to consider one effect from life time horizon and the cost from a one year horizon.**

We agree with the reviewer that our explanation of how we estimated health outcomes and associated costs prevented by COVID-19 interventions was confusing. We assume the effect of the intervention is to reduce incidence and severe disease over one year – that is, immunity due to antivirals or booster doses only lasts one year. However, the cases of severe disease prevented over this one year are associated with longer-term health benefits. We have rewritten this paragraph in our methods to provide a clearer explanation:

*(Excerpt from the manuscript – line 95)*

*We estimated the reduction of severe disease by booster doses and oral antivirals over the course of one year, aligning with existing economic evaluation of COVID-19 interventions [9].* *We evaluated the impact of these interventions by including the ongoing costs and morbidity associated with cases of severe disease prevented within this year, discounting by 3%. Estimating the reduction in severe disease by oral antivirals over one year was appropriate because the effects of oral antivirals are short-term and localised to the individual receiving antivirals. Estimating the reduction in severe disease by booster doses over one year was conservative but appropriate because the increased protection against severe outcomes provided by booster doses compared to previous vaccination is expected to wane after one year [10].*

**Comment 2: Table 2 parameter estimation “Cost per extra day hospital length of stay” is not clear.**

We have amended this parameter to read “cost per additional inpatient day” to improve clarity.

**Comment 3: Probabilities that are used in the model are not described in table. Please provide the probabilities that are used to populate the SEIR model, all parameters used to populate the model should be presented on one table.**

We have moved the probability distributions of parameter values for the cost-effectiveness analysis from the supplementary material to the manuscript.

*(Excerpt from the manuscript – line 197)*

***Table 1. Parameters informing booster dose and oral antiviral program costs.*** *All costs reported in 2022 United States Dollars. Probability distributions described where parameter values not fixed within simulations.*

| ***Parameter*** | ***Component*** | ***Estimate*** | ***Source*** |
| --- | --- | --- | --- |
| *…* |  |  |  |
| *Operational costs (vaccine)* | *All other costs including cost of personnel, training, transport etc.* | *$2.85*  *Sensitivity analysis: $0.21-$13.04*  *Distribution: Lognormal(meanlog = 0.80, sdlog = 0.70)* | *Meta-analysis of routine immunisation delivery costs in low- and middle-income countries [17]* |
| *…* |  |  |  |
| *Operational costs (antiviral)* | *Price per outpatient visit for individual to be prescribed oral antivirals* | *$4.36 – Fiji*  *$2.57 – Indonesia*  *$3.91 – Papua New Guinea*  *$1.83 – Timor-Leste*  *Distributions provided in Table 2* | *WHO CHOICE estimates for the cost per outpatient visit to a health centre with no beds [40]* |

*(Excerpt from the manuscript – line 246)*

***Table 2.*** ***Parameters estimating healthcare costs associated with COVID-19.*** *All costs reported in 2022 United States Dollars. Lognormal distributions provided with meanlog and sdlog; normal distributions provided with mean and standard deviation.*

| ***Component*** | ***Estimate*** | ***Sampling distribution*** | ***Source*** |
| --- | --- | --- | --- |
| *Cost per hospital admission* | *$7,999.82 – Fiji*  *$5,847.48 – Indonesia*  *$3,858.55 – Papua New Guinea*  *$3,081.46 – Timor-Leste* | *Normal(7,999.82, 5.7)*  *Normal(5,847.48,4.1)*  *Normal(3,858.55,2.2)*  *Normal(3,081.46,2.2)* | *[41]* |
| *Cost per outpatient visit* | *$4.36 – Fiji*  *$2.57 – Indonesia*  *$3.91 – Papua New Guinea*  *$1.83 – Timor-Leste* | *Lognormal(1.24,0.689)*  *Lognormal(0.69,0.72)*  *Lognormal(0.68,1.13)*  *Lognormal(0.64,0.40)* | *[40]* |
| *Proportion of non-hospitalised symptomatic individuals accessing healthcare* | *81.6% – Fiji*  *70.0% – Indonesia*  *70.0% – Papua New Guinea*  *76.6% – Timor-Leste* | *Fixed* | *[51-53]* |
| *Reduced length of stay of patients who received oral antivirals* | *0.784 days* | *Normal(0.784,0.386)* | *[54]* |
| *Cost per additional inpatient day* | *10% of cost per hospital admission* | *Normal(0.10,0.01)* | *[41]* |

The probability distributions underlying the transmission model are described in its associated published paper, and clearly documented in the *02_stochastic_outcomes_projections* folder of our linked GitHub repository. These probability distributions cannot be provided as a table as they involve sampling from many observational estimates, imputing values based on the values sampled, and then adjusting estimates using other parameters with their own associated uncertainty.

For example, to estimate the setting-specific likelihood of developing severe outcomes we first sample from observational estimates for age-specific severity. We calculate relative risk by age. We adjust these estimates from the observational study’s age groups to our model age groups. Then, we sample from setting-specific estimates for severity to calculate age- and setting-specific severity. This value is for wild-type COVID-19, so we sample from a severity multiplier to adjust estimates to Omicron.

**Comment 4: Line 254: please review the distribution used for the parameters. “Commonly used distributions In economic modelling are symmetrical, such as the normal distribution, often used for parameters such as population age and intervention effectiveness (e.g. relative risk reduction), or skewed, such as gamma or lognormal, for ratios or for parameters such as costs which cannot be negative.”**

We have reviewed the distributions used for parameters. As the reviewer suggest, we have used skewed lognormal distributions for operational costs of delivering vaccines, and outpatient costs of treating patients. We have used symmetrical normal distributions for estimates of antiviral effectiveness in reducing hospital lengths of stay. The one instance in which we use a normal distribution for a cost is for cost per hospital admission, <0.00001 % of the probability distributions for this parameter is greater than zero.

**Comment 5: Please, explain the results with numbers. See my previous comment from last review. “The result section needs to have numbers reported in it. Example: when doing probabilistic sensitivity analysis, the authors should report the probability of being cost-effective with percentage.”**

As suggested by the reviewer, we have reported numbers in the results section where they are relevant. We have intentionally not included probabilities equal to 100% to ensure the readability of the text. To make this clear, we have included the following sentence in the manuscript:

*(Excerpt from the manuscript – 261)*

*We have reported the probability of interventions being cost-effective with percentages in the results text in all cases where the estimated probability is not 100%.*
