# Supplementary material for: Cost-effectiveness analysis of COVID-19 booster doses and oral antivirals: Case studies in the Indo-Pacific
Source: PLoS One. 2024 Sep 30;19(9):e0294091. doi: 10.1371/journal.pone.0294091 (PMC11441647; doi:10.1371/journal.pone.0294091)
Supplement: Attachment — Submitted filename: 2024_09_12 Response to Reviewers.docx [file pone.0294091.s005.docx]

We thank the reviewers for providing feedback on our revised manuscript. We have included further detail in response to their suggestions.

**Reviewer #1**

**Generally, the paper is worth publishing, with a few suggestions for minor changes.**

**Comment 1: The study evaluates the cost-effectiveness of COVID-19 booster doses and oral antivirals in the Indo-Pacific. However, the countries covered are Fiji, Indonesia, Papua New Guinea, and Timor-Leste. I am not sure exactly how big part from the Indo- Pacific they represent and would appreciate comment on this.**

We have revised the title of the paper to “Cost-effectiveness analysis of COVID-19 booster doses and oral antivirals: case studies in the Indo-Pacific” to acknowledge that our four settings do not represent the whole region.

**Comment 2: Minor. Methodologically, CEA, per se, is associated with well-acknowledged shortcomings, such as stochastic uncertainty, heterogeneity and treatment stratification, representation uncertainty, misinterpretation of censored data, list not exhaustive. Concerns regarding QALYs have been previously described in many articles, including a 2009 supplement to Value in Health. These limitations are not addressed in the relevant section of the paper.**

We have read the 2009 supplement in Value in Health suggested by the reviewer and considered the limitations of cost-effectiveness analysis they list:

- Stochastic uncertainty – We explored the effects of stochastic uncertainty by conducting and visualising probabilistic sensitivity analysis.
- Heterogeneity and treatment stratification – To capture variations in how different subgroups respond to interventions, our model includes age-specific estimates for vaccine effectiveness and infection-derived immunity. Additionally, the effectiveness estimates for antivirals are derived from observational studies specific to the subgroups in which we assumed they were used.
- Representation uncertainty – We have acknowledged the uncertainty regarding how well the model reflects real-world decision-making scenarios by adding a sentence to the discussion (line 448).
- Misinterpretation of censored data – We recognise uncertainty in outcomes such as waning immunity and ongoing morbidity, given that COVID-19 is a relatively new pathogen. This limitation is discussed in the manuscript, particularly in our use of a one-year time horizon in line with other published analysis.
- Concerns regarding QALYs – We acknowledge the limitations of QALYs, particularly their inability to account for social values, such as the cultural variation in valuing the health of older adults. We have included a sentence in the discussion stating that cultural values will affect a decision maker’s willingness to pay per QALY (line 450).

**Comment 3: Minor. Incremental net monetary benefit (INMB) can be presented as an alternative to the ICER. The INMB uses the WTP threshold to convert the QALY into a monetary value. Would the authors consider inclusion of INMB or at least comment its absence?**

We chose to present incremental cost-effectiveness ratios (ICERs) instead of estimates of incremental net monetary benefit (INMB) due to a lack of data on the value of quality-adjusted life years (QALYs) in our study settings. Surveys that provide empirical estimates of QALYs are more common in high-income countries. In our manuscript, we highlight the significant uncertainty around the willingness to pay (WTP) for QALYs saved by COVID-19 interventions, especially given the current landscape of increased immunity against COVID-19. Ultimately, we decided that making another assumption in our analysis would not improve the clarity of our findings.

**Comment 4: Minor. Authors are encouraged to elaborate a bit more about the “antivirals”. Indeed, Pfizer is mentioned /Paxlovid?/, are there other/s? It is important to avoid drugs subject to off- label use as well as to note the substantial price differences observed in the access provided during the pandemic period on a country-to-country basis.**

Our manuscript presents results for nirmatrelvir-ritonavir (brand name: Paxlovid) and we provide additional results for molnupiravir (brand name: Lagevrio) in the Supplementary Material. We have made this explicit earlier in the methods and when describing the three reference prices.

*(Excerpt from the manuscript – line 88)*

*We assumed the use of nirmatrelvir-ritonavir and provided additional results for molnupiravir in the Supplementary Material and R Shiny.*

*(Excerpt from the manuscript – line 173)*

*We assessed the cost-effectiveness of oral antivirals at three schedule prices: a high-income country reference price of US$530 [26], a middle-income reference price of US$250 [27], and the anticipated low generic price of US$25 [28] for nirmatrelvir-ritonavir.*

**Comment 5: Minor. In the PSA authors mentioned 1 000 times. Kindly rephrase to 1 000 Monte Carlo simulation, if this you meant.**

We have made this suggested edit throughout the manuscript and supplementary material when referring to the probabilistic sensitivity analysis.

**Comment 6: Minor. I noticed self- citations in references. Authors are encouraged to explain and act on it.**

This cost-effectiveness analysis builds off a mathematical model of COVID-19 transmission developed in a previous paper. Citing this work enables the reader to access further detail on the methods and epidemiological outputs of the model. We have made it explicit in the manuscript that we are citing our previous work:

*(Excerpt from the manuscript – line 83)*

*We quantified the epidemiological impacts of COVID-19 booster doses and oral antivirals in a previous mathematical modelling paper [5].* *In this paper, we estimated the cost of administering COVID-19 booster doses, the cost of delivering COVID-19 oral antivirals, and the costs averted by either intervention using publicly available data.*

*(Excerpt from the manuscript – line 110)*

*We have described the details of the deterministic COVID-19 transmission model and stochastic severe outcome projections in previous papers [5, 11], and a summary is provided in S1 Underlying Dynamic Transmission Model.*
